# Disclosing the Impact of Carcinogenic SF3b Mutations on Pre-mRNA Recognition Via All-Atom Simulations

**DOI:** 10.3390/biom9100633

**Published:** 2019-10-21

**Authors:** Jure Borišek, Andrea Saltalamacchia, Anna Gallì, Giulia Palermo, Elisabetta Molteni, Luca Malcovati, Alessandra Magistrato

**Affiliations:** 1 CNR-IOM-Democritos National Simulation Center c/o SISSA, 34136 Trieste, Italy; jure.borisek@ki.si; 2 National Institute of Chemistry, 1000 Ljubljana, Slovenia; 3 International School for Advanced Studies (SISSA), 34136 Trieste, Italy; asaltala@sissa.it; 4 Department of Hematology, IRCCS S. Matteo Hospital Foundation, 27100 Pavia, Italy; anna.galli@unipv.it (A.G.); luca.malcovati@unipv.it (L.M.); 5 Department of Bioengineering, University of California Riverside, Riverside, CA 92521, USA; gpalermo@engr.ucr.edu; 6 Department of Molecular Medicine, University of Pavia, 27100 Pavia, Italy; elisabetta.molteni@unipv.it

**Keywords:** RNA, molecular dynamics, spliceosome, splicing

## Abstract

The spliceosome accurately promotes precursor messenger-RNA splicing by recognizing specific noncoding intronic tracts including the branch point sequence (BPS) and the 3’-splice-site (3’SS). Mutations of Hsh155 (yeast)/SF3B1 (human), which is a protein of the SF3b factor involved in BPS recognition and induces altered BPS binding and 3’SS selection, lead to mis-spliced mRNA transcripts. Although these mutations recur in hematologic malignancies, the mechanism by which they change gene expression remains unclear. In this study, multi-microsecond-long molecular-dynamics simulations of eighth distinct ∼700,000 atom models of the spliceosome Bact complex, and gene sequencing of SF3B1, disclose that these carcinogenic isoforms destabilize intron binding and/or affect the functional dynamics of Hsh155/SF3B1 only when binding non-consensus BPSs, as opposed to the non-pathogenic variants newly annotated here. This pinpoints a cross-talk between the distal Hsh155 mutation and BPS recognition sites. Our outcomes unprecedentedly contribute to elucidating the principles of pre-mRNA recognition, which provides critical insights on the mechanism underlying constitutive/alternative/aberrant splicing.

## 1. Introduction

**Figure 1:**
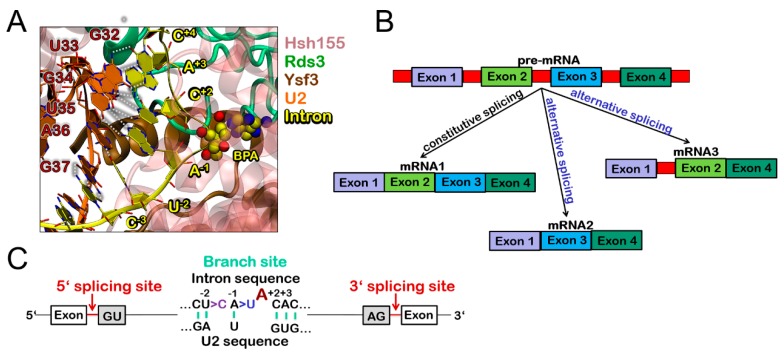
(**A**) Intron/U2 double helix at branch point site (BPS), as trapped in the B^act^ cryo-Electron microscopy (EM) structure (Protein Data Bank (PDB) code 5GM6). The bulged adenine (**A**) at the BPS is shown in van der Waals (VDW) spheres and labelled as branch point adenosine (BPA). The surrounding Hsh155, Rds3, and Ysf3 proteins are depicted as pink, green, and brown cartoon representations, respectively. Hydrogen (H)-bonds of base-pairs between intron and U2 are highlighted as white dashed lines. (**B**) Simplified representation of constitutive and alternative splicing. Large boxes depict the exons, while small red rectangles refer to introns. Constitutive splicing (resulting in mRNA1) is schematically presented as exons ligated in the same order in which they appear in pre-mRNA. In alternative splicing, one pre-mRNA can be spliced in different transcripts (mRNA2, mRNA3), encoding for different proteins. (**C**) Key intron recognitions sites. BPA at the branch site is indicated with a dark red letter. Replacement either of A to U at position -1 or of U to C at position-2 disrupts the intron-U2 base-pairing, which results in a non-consensus BPS. The intronic sequence at the 5’ splicing site starts with the highly conserved GU nucleotides and ends at the 3’ splicing site with the conserved AG nucleotides.

In eukaryotic cells, the exons, which are the coding regions of a newly transcribed precursor messenger RNA (pre-mRNA), are interspersed by non-coding regions, the introns, which have to be dismissed to produce mature mRNA before protein translation occurs. Introns removal from a nascent RNA transcript is, therefore, a pivotal step of gene expression and regulation. In eukaryotes, this process is carried out by the repeated assembly of the spliceosome (SPL), which is a majestic multi mega Dalton ribonucleic-protein machine comprising more than 100 proteins and five small nuclear RNAs (snRNAs: U1, U2, U4, U5, and U6) [1,2]. These latter congregate, through an entangled network of interactions, into five distinct small nuclear ribonucleoproteins (snRNPs), i.e., the U1, U2, U3, U4, and U5 snRNPs. The SPL processes long and diverse RNA transcripts with single nucleotide precision via the formation of eight distinct complexes, at every splicing cycle (A, B, B^act^, B^*^, C, C^*^, P, and ILS). Splicing fidelity is achieved via the recognition of consensus sequences near the 5′ and 3′ ends of introns, known as 5′ and 3′ splice sites (5’SS and 3’SS, respectively). In detail, a conserved GU sequence at the 5’SS is bound by U1snRNP, upon A complex assembly, while, the appropriate 3′ SS is selected by the U2 snRNP upon B complex formation. 3’SS selection takes place via the recognition of short RNA regions such as the branch point sequence (BPS), the polypyrimidine tract, and the AG dinucleotide at the intron-exon junction. Among these key sequences the BPS, containing a conserved branching point adenosine (BPA) at the branch site (BS), is recognized by the Hsh155 (yeast)/SF3B1 (human) protein in the B^act^ complex (Figure 1A,B).

Two sequential trans-esterification reactions, which are mediated by two Mg^2+^ ions [3,4,5], lead to intron excision and exon ligation, promoting pre-mRNA maturation. Constitutive splicing occurs via snipping of introns and stitching of exons in the same order in which they appear in pre-mRNA. Alternative splicing is, instead, a divergence from this preferred sequence. In this latter case, distinct exons and intron/exon junctions may be alternatively employed (i.e., some exons may be skipped and/or introns may be retained), which produces different mRNA splicing products from the same primary transcript (Figure 1B). As a result, multiple protein isoforms are created from a single gene [6]. The possibility and amount of alternatively spliced genes increase with the complexity of the organisms, which is the hallmark of higher eukaryotes.

In order to promote alternative splicing, the SPL must recognize and process non-consensus intronic sites (i.e., sites differing from the consensus ones by one nucleobase), while still removing the introns with extreme fidelity. The latest evidence suggests that Hsh155 (yeast)/SF3B1 (human) protein, part of the SF3b splicing factor, may be in charge of decreasing the specificity of SPL toward BPS recognition, which enables it to bind and process consensus and non-consensus BPS variants (cBPS and ncBPS, respectively) [7] and, thus, being crucially involved in the regulation of constitutive or alternative splicing. Mounting evidences pinpoint to specific SPL mutations, which affect proper intron recognition, as responsible for dysregulated alternative splicing. These lead to the production of aberrant mRNA transcripts [8,9], which become key drivers of major human diseases [2,10,11,12]. In this respect, large-scale genomic studies indicate that mutations of the Hsh155/SF3B1 protein recur in hematologic malignancies (i.e., myelodysplastic syndromes (MDS) [13], chronic lymphocytic leukemia [14], and chronic myelomonocytic leukemia [15], and less commonly in solid tumors [7]). Bioinformatics analyses revealed that Hsh155/SF3B1 mutations are involved in aberrant splicing by altering BPS selection [7,16,17].

Recent cryo-EM structures of the catalytically activated B^act^ SPL complex from yeast *Saccharomyces cerevisiae* [18], from humans [2,19], and a crystal structure of the human SF3b [20,21,22], have clarified the molecular details of the Hsh155/SF3B1 protein and elucidated that Hsh155/SF3B1 directly contacts the intron/U2 snRNA duplex, which stabilizes the bulged BPA. The Hsh155/SF3B1 mutations implicated in hematologic cancers, map on the C-terminal part of its HEAT (huntingtin elongation factor 3 protein phosphatase 2A, target of rapamycin 1) -repeat structure. This region interacts with the intron between the BPS and 3’SS recognition sites. In spite of its pivotal importance, the mechanisms by which Hsh155 mutations affect intron selection and change gene expression remain elusive. The intricacies of this mechanism further increase considering that MDS Hsh155 variants have been recently demonstrated to only mis-regulate the splicing of introns containing a ncBPS [7].

To unravel the functional dynamics of the B^act^ complex and assess the impact of the selected Hsh155/SF3B1 mutations on splicing fidelity at an atomic level of detail, we employed cumulative multi-microsecondlong molecular dynamics (MD) simulations and gene sequencing. We focused on eight distinct model systems of the yeast B^act^ complex exploring the impact of cBPS, two distinct ncBPS (A-1U and U-2C), either taken singularly or in combination with two pathogenic K335E or N295D Hsh155 variants recurrently expressed in MDS. Moreover, we also include the non-disease causing L378V Hsh155 isoform, annotated here on the basis of gene sequencing studies of SF3B1 and public database analysis. Besides confirming the leading role of Prp8 in orchestrating the motion of the distinct protein/snRNAs components of B^act^, our outcomes stunningly disclose that (i) Hsh155 can bind/recognize both cBPS and ncBPS via an opening/closing motion of its super-helical structure, in line with experimental evidence [7,21], (ii) the peculiar HEAT-repeat structure of Hsh155 allows a cross-talk between the pathogenic mutation and the distal BPS recognition site by enhancing the opening/closing spring-like motion of Hsh155, and/or by affecting intron binding, only when an non-consensus (nc) BPS is present. As a result, these mutants may adversely affect splicing by weakening ncBPS binding, possibly facilitating its release, and inducing the recruitment of a cryptic 3’SS (a site that would be not spliced in non-pathologic conditions), in line with experimental suggestions [7]. Hence, our findings provide fundamental and unprecedented insights on the mechanisms regulating the subtle balance among constitutive/alternative/aberrant splicing.

## 2. Materials and Methods

### 2.1. Structural Models

We built eight different models starting from the yeast *Saccharomyces cerevisiae* B^act^ cryo-electron microscopy (EM) structure solved at the average resolution of 3.5 Å (PDB entry 5GM6) [18], which, in the central part, reaches 2.8–3.2 Å resolution. This structure captured the SPL prior to the first splicing step. As in our previous study [23], our models account for the central and best resolved portion of the cryo-EM structure. Namely, they comprise (i) Prp8, the most important and conserved protein of the SPL, and (ii) the SF3b complex proteins Rds3, Ysf3, and Hsh155 (corresponding to PHF5A, SF3B5, and SF3B1 in human, respectively). Moreover, we include (iii) five RNA filaments (U2, U5, and U6, intron and exon), and (iv) four Mg^2+^ and three Zn^2+^ ions (Table S1). Other components of B^act^ system were either incomplete or solved with resolution not appropriate for atomic-level simulations. Thus, they were not included in the models. In this case, we have chosen not to rebuild large portions of proteins plagued by the presence of multiple and large gaps and, in particular, RNA filaments. This was done to avoid the incurrence of unpredictable results, which may arise from the well-known issues of the RNA force filed [24,25], which also affect protein-RNA interactions and the limited accuracy of RNA modeling tools to predict a secondary structure. In addition, the model has been selected considering the proteins that surround the portion of the RNA filaments relevant for this study, and to find a compromise between system size and accuracy. This strategy has been successfully employed in a previous simulation study of the SPL, in which the selection of models of different sizes confirmed the reliability of our approach [23].

De novo model building, as implemented in Modeler 9, version 16 [26], was used to reconstruct missing loops of Prp8 and Hsh155. The generated loops were first selected among 50 models, according to the DOPE score [27], and, subsequently, evaluated through an accurate visual inspection. The model corresponding to the wild-type sequence of the considered proteins and RNA filaments is referred as **Bact**. Starting from this structure (i) the K335E mutant, involved in MDS, was inserted in Hsh155 resulting in a model, denoted as **^K335E^Bact**, (ii) next, the nucleotide A at position -1 with respect to the BPA was mutated to U, generating the first non-consensus BPS sequence. The resulting model is referred to as **Bact_A-1U_** (ii). Next, (iii) U at position -2 with respect to the BPA was mutated to C by generating the second ncBPS. Either ncBPSs were introduced in **^K335E^Bact**, which resulted in the (iv) **^K335E^Bact_A-1U_** and (v) **^K335E^Bact_U-2C_** models. Another Hsh155 pathogenic mutation N295D was chosen and considered with both a consensus BPS and ncBPS A-1U named as (vi) **^N295D^Bact** and (vii), **^N295D^Bact_A-1U_**, respectively. Lastly, we selected the non-pathogenic mutation L378V and inserted it in Hsh155 along with the ncBPS A-1U sequence resulting in (viii) the **^L378V^Bact_A-1U_** model. All mutations were inserted in the models by using the leap module of Ambertools 16 [28]. Due to the large size of the investigated system, we limited the number of models to those necessary to inspect relevant differences between pathological and non-pathological variants. This choice led, nevertheless, to the simulations of eight distinct 700,000 atom models.

### 2.2. Molecular Dynamics (MD) Simulations

Molecular Dynamics simulations were performed with a Gromacs2016 software package [29]. The AMBER-ff12SB force field (FF) [29] was employed for proteins [30], while the ff99+bsc0+χOL3 FF was used for RNAs [31], since these are the most validated and recommended FFs for protein/RNA systems [32], and showed reliable results in our previous simulation study of the intron lariat system (ILS) of SPL [23] and in other RNA simulation studies [33,34,35]. Mg^2+^ ions were described with the non-bonded fixed point charge FF due to Åqvist [36], since it was shown to properly describe binuclear sites [4,37]. Na^+^ ions parameters were taken from Joung and Cheatham [38], while Zn^2+^ ions were modeled with the cationic dummy atoms approach developed by Pang [39], such as in our previous study [23]. The system was embedded in a 10 Å layer of TIP3P [40] water molecules leading to a box of 169 × 161 × 262 Å^3^, containing four Mg^2+^ ions, three Zn^2+^, and 167 Na^+^ counter ions counting up to 666,641 atoms. Due to the relevance and the impact of metal ions on the structural properties of RNA, we have also performed a control simulation by reproducing the physiological KCl ionic strength [41]. The topologies were built with Ambertools 16 [28] and were, subsequently, converted in a GROMACS2016 format using the software acpype [42]. 

In all simulations, we have used a slow equilibration protocol, described previously [23], and recommended for protein/RNA MD simulations [32]. Namely, the systems went initially through a soft minimization using a steep descent algorithm with a force convergence criterion set to 1000 kJ mol^−1^ nm^−1^. Then, the models were smoothly annealed from 0 to 300 K with a temperature gradient of 50 K every 2 ns and for a total of 12 ns. In this phase, only water molecules and ions (Na^+^, K^+^ and Cl^−^) were allowed to move, while the rest was subjected to harmonic position restraints with a force constant of 1000 kJ/mol nm^2^. Once the temperature was raised up to 300 K, 20 ns of isothermal-isobaric ensemble (NPT) simulations were conducted to stabilize the pressure to 1 bar by coupling the systems to a Berendsen barostat [43], which imposed the same restraints used in the heating phase. Temperature control at 300 K was achieved by a stochastic velocity rescaling thermostat [44]. Subsequently, the barostat was switched to Parrinello-Rahman [45,46] and the position restraints on proteins and RNAs were restricted only to the backbone atoms. These were gradually decreased in three consecutive steps of 30, 10, and 10 ns each, during which the force constant was set to 1000, 250, and 50 kJ/mol nm^2^, respectively. Lastly, after an attentive equilibration protocol of ~80 ns, all the restraints were released and the production runs were performed for ~500 ns (for a total of ~580 ns) for each of the subjected models. Productive MD simulations were performed on NPT ensemble using periodic boundary conditions. The LINCS algorithm [47] was used to constrain the bonds involving hydrogen atoms and the particle mesh Ewald method [48] to account for long-range electrostatic interactions with a cutoff of 12 Å. An integration time step of 2 fs was employed in all simulations, as used in other studies of similar systems [35,49]. For **Bact**, we performed three independent replicas of simulations starting from different initial velocities to check the convergence of our results. Furthermore, we also performed additional 580 ns length MD simulations on **Bact_A-1U_**, **^K335E^Bact**, **^K335E^Bact_A-1U_**, **^K335E^Bact_U-2C_**, **^L378V^Bact_A-1U_, ^N295D^Bact**, and **^N295D^Bact_A-1U_** models, reaching an overall simulation time of 5.8 µs (10 × 580 ns). We, additionally, performed 300 ns-long MD simulation on Bact in the presence of 0.15 M KCl (hereafter, **Bact_KCl_**) to control the impact of the type of ions and of the ionic strength on the system [41].

The trajectories were inspected and analyzed with the VMD software [50]. All analyses, including root mean square deviation (RMSD), radius of gyration (Rg), root mean square fluctuations (RMSF), principal component analysis (PCA), and the calculation of the cross-correlation matrices were done with the cpptraj module of Ambertools 16 [28] and with Gromacs2016 [29] suite on the stripped trajectories without water and counter-ions. For the hydrogen (H) bonds, the analysis have been conducted with the cpptraj module of Ambertools 16 using a cutoff of 3.3 Å between acceptor and donor heavy atoms with the maximum angle of 145°. Analyses were performed on the last 500 ns of trajectory. We also monitored the convergence of the properties on the **Bact** model over the last 380 ns (see Supporting Information, Figures S1 and S12).

### 2.3. Principal Component Analysis

Principal component analysis was performed with the cpptraj module of Ambertools 16 [28] to extract the essential dynamics of the distinct Bact models. PCA can capture the large-scale collective motions occurring in biological molecules undergoing MD simulations, which provides information on the major conformational changes occurring along the MD trajectories [51,52]. The essential motions of proteins and RNAs have been pictured starting from the mass-weighted covariance matrix of the Cα and P atoms, respectively. The covariance matrices were built from the atoms position vectors upon an RMS-fit to the reference starting configuration of the MD production run in order to remove the rotational and translational motions, as described previously [23]. Briefly, the eigenvectors with the largest eigenvalues correspond to the direction of the most relevant motions sampled during the simulation, which is also referred to as principal components (PCs). By projecting the displacement vectors of each atom along the trajectory onto the eigenvectors, it is possible to reduce the dimensionality and the noise inherent in a trajectory, by obtaining only the most relevant motions. The cumulative variance accounted by the PCs was calculated for all models. The Normal Mode Wizard plugin [53] of Visual Molecular Dynamics (VMD) program was used to visualize the essential dynamics along the principal eigenvectors and to draw the arrows highlighting their direction. Calculations have been performed on all subjected systems even though, in the figures, we only report the essential dynamics of Hsh155 for clarity reasons. 

### 2.4. Cross Correlation Matrix and Correlation Scores

The cross-correlation matrices (or normalized covariance matrices) based on the Pearson’s correlation coefficients (CCij) were calculated with the cpptraj module of Ambertools 16 [28] from the previously obtained covariance matrices. In order to make the correlation matrices clear at first glimpse, we have accumulated the correlation for each SPL component, by means of correlation scores (CSs) between each SPL component and all the others. This approach, already introduced to decrypt the correlation pattern observed in complex biomolecules [23,34,54,55,56], results in a simplified variant of the CCij matrix [34,54]. Due to the large size of some proteins and to better dissect their role in a simplified version of the cross-correlation matrix, we separately considered the Prp8 domains and Hsh155 HEAT-repeats. Next, each sum of CSs of pair proteins/domains was divided by the product of the number of residues belonging to this pair of proteins/domains, which obtains a correlation density per each area. The obtained scores were plotted as matrices, showing in a simplified and clear manner the type of correlated motions between each pair proteins/domains. Switch regions were determined based on both a sudden change in the cross-correlation matrix (from positive to negative correlation) and by PCA analysis as in the hinge region. No vector (movements) is associated with the atoms constituting the pivot of the observed movement.

### 2.5. Electrostatic Calculations

Electrostatic calculations were performed with the Adaptive Poisson-Boltzmann Solver (APBS) software on selected proteins of the **Bact**, **Bact_A-1U_**, **^K335E^Bact**, **^K335E^Bact_A-1U_**, **^K335E^Bact_U-2C_**, **^L378V^Bact_A-1U_, ^N295D^Bact**, and **^N295D^Bact_A-1U_** models considering representative frames of simulation extracted from the cluster analysis [57]. To monitor the effect of the ionic strength on electrostatic properties, the previously mentioned procedure was also applied to the **Bact_KCl_** model. The selected geometries of the models were converted to the pqr format with PDB2PQR software [58,59]. The APBS assesses electrostatic properties of proteins by solving the Poisson-Boltzmann equation [60]. APBS calculations were carried out using the Linearized Poisson-Boltzmann Equation (LPBE) in Chimera software [61] with the following settings: surface density of 10.0 points/Å2, solvent radius of 1.4 Å, system temperature of 298.15 K, solute dielectric constant of 2.0, and solvent dielectric constant of 78.54 with a smoothed molecular surface.

### 2.6. SF3B1 Sequencing and Variant Annotation

SF3B1 hot-spot exons [13,14,15,16] were analyzed using an amplicon-based next generation sequencing panel (TruSight Myeloid Sequencing Panel, Illumina, San Diego, CA, USA). Probes were hybridized to 250 ng of gDNA, and an extension-ligation reaction extended across the selected region, which was followed by a ligation step. The resulting templates were amplified by PCR and two unique library specific indexes were incorporated. The resulting libraries were normalized to the same concentrations using a fluorescence-based quantification procedure that enables pooling of libraries. Pooled DNA libraries were loaded onto the cBot System for cluster generation followed by 2 × 250 paired-end sequencing on a HiSeq2500 sequencer (both from Illumina). Functionally annotated variants were then classified based on the information retrieved from public databases (dbSNP, 1000genome, and ESP6500), the expected germ line allele frequency, and the information derived from the literature, the Catalog of Somatic Mutations in Cancer (COSMIC; http://cancer.sanger.ac.uk/cancergenome/projects/cosmic), and an in-silico prediction effect. Variant allele frequency was calculated as the number of variant reads divided by the total reads.

## 3. Results

### 3.1. Molecular Framework of BPS Recognition in the Wild-Type Model

**Figure 2:**
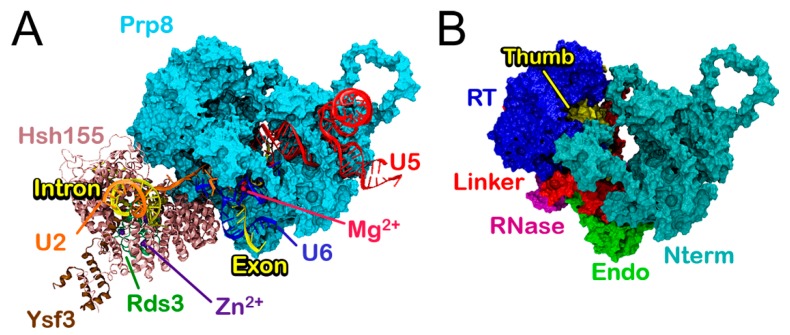
Spliceosome **Bact** model built on the yeast *Saccharomyces cerevisie* B^act^ cryo-EM structure (Protein Data Bank code: 5GM6) [18]. (**A**) Proteins (Hsh155 (light pink), Rds3 (green) and Ysf3 (brown) and RNAs (U2 (orange), U5 (red), U6 (blue), intron, and exon (yellow) are depicted as cartoons, whereas Prp8 is shown as a cyan surface. Mg^2+^ and Zn^2+^ ions are represented as light-red and violet spheres, respectively. (**B**) Domain subdivision of Prp8 into Nterm (dark cyan), RT (blue), Thumb (yellow), Linker (red), Endo (light green), and RNase (purple) shown as surfaces.

We initially inspected the structural traits of the 700,000 atoms wild-type model, hereafter referred as **Bact**. This model, extracted from the yeast *S. cerevisiae* B^act^ structure (PDB ID 5GM6) [18] comprising five proteins (Prp8, Hsh155, Rds3, Ysf3) and five RNA filaments (U2, U5, U6, intron and exon), was relaxed by performing three 580 ns long MD simulations using AMBER-ff12SB force field (FF) for proteins [30] and ff99+bsc0+χOL3 FF for RNAs [31]. In this structure preceding the first splicing step, the 5’-exon is bound to U5 while the 5’SS and the BPS are recognized by U6 and U2 snRNAs, respectively. Hsh155, part of the U2 snRNP, binds/recognizes the BPS and the flanking sequence of the intron (Figure 1A and Figure 2).

The structural convergence of this model was reached within 580 ns for all replicas (Figure S1). The fragile structural motif of the catalytic site retains structural stability along the whole simulation. Namely, the RNA triplex consisting of A59, G60, and C61 nucleotides from U6, maintains its canonical base-pairing with U23, C22, and G21 from U2 snRNA, and non-canonical base-pairing with A53, G52, and U80 from U6, respectively (Figure S2). This environment defines a nest able to host four Mg^2+^ ions, among which the catalytic ones. The distance between the BPA and scissile G-G bond at 5’SS remains approximately 48 Å, which is in line with the not yet catalytically competent nature of the B^act^ complex [18]. The BPA is engulfed in a pocket formed by the HEAT-repeats (H)15-16 of Hsh155, where it is stabilized by H-bonds and hydrophobic interactions with Q747, R775, K818, and Y826 (Figure S3), while the intron bases, down-stream and up-stream the BPA, base pair with U2 snRNA. An analysis of the electrostatic potential (Figures S4 and S5) elucidates that the Rds3-Hsh155 positively charged interface traps the intron. This was also observed in the **Bact_KCL_** model, which mimics the physiological ionic strength (Figure S6).

### 3.2. Functional Dynamics of the Bact Model

**Figure 3:**
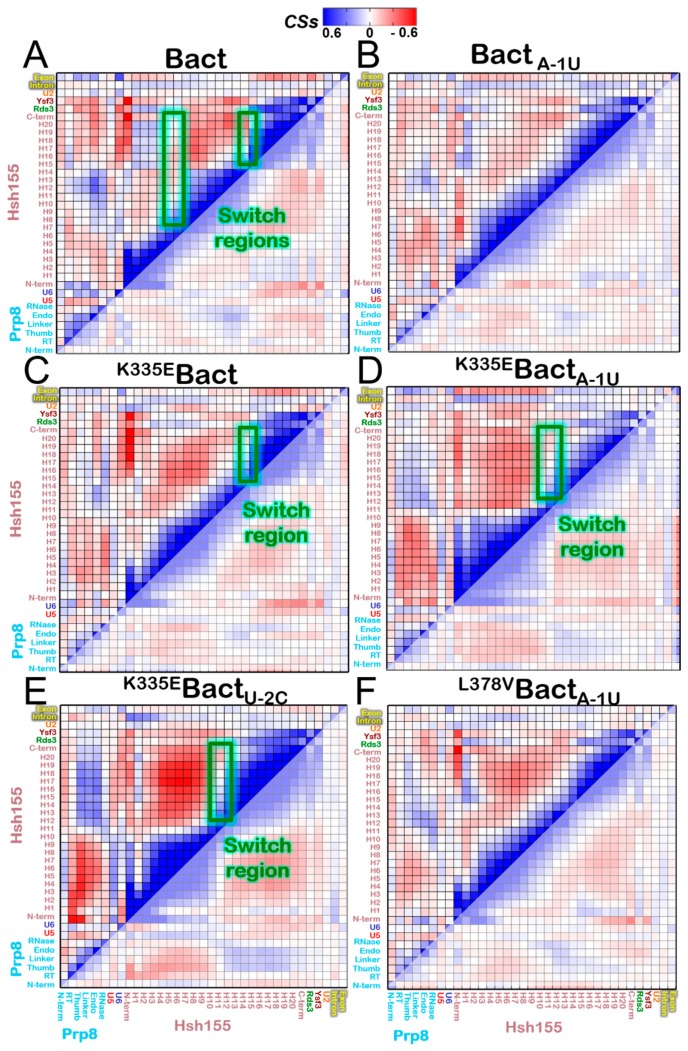
Cooperative motion underlying the functional dynamics of the distinct Bact models investigated. Per-residue Pearson’s coefficients (CCs) cross-correlation matrix is derived from the mass-weighted covariance matrix calculated over the last 500 ns of classical molecular dynamics trajectories. CCs values range from −1 (red, anti-correlated motions) to +1 (blue, correlated motions) are summed for each pair of considered spliceosome (SPL) proteins/domains, and normalized to provide density correlation scores (CSs). No cutoff has been applied here on the CCs selection. Instead, CSs are reported in the range from −0.6 to 0.6 for clarity reasons. We remark that, with this choice, there are elements out of the range (i.e., the element with value = 1.0 has the same color as that with 0.6). The Hsh155 is split by HEAT (huntingtin elongation factor 3 protein phosphatase 2A, target of rapamycin 1)-repeats and Prp8 is divided by domains. In green are encircled the Hsh155 regions where a switch between a positive and a negative correlation occurs. Depicted are CSs of (**A**) **Bact**, (**B**) **Bact_A-1U_**, (**C**) **^K335E^Bact**, (**D**) **^K335E^Bact_A-1U_**, (**E**) **^K335E^Bact_U-2C_**, and (**F**) **^L378V^Bact_A-1U_** models. Bact models are labeled reporting the Hsh155 and BPS mutations as left superscript and right subscript, respectively. Protein names and their domains are labelled on the bottom and left of the matrix.

Consistently with our previous study [23] and with the wealth of cryo-EM structures solved to date, our MD simulations assign to Prp8 a leading role in modulating the functional dynamics of all distinct B^act^ components considered in our model. This was revealed by the cross-correlation matrices (or normalized covariance matrices) based on the Pearson’s correlation coefficient (CCij), which allows to qualitatively pinpoint the linearly coupled motions between the pair of residues along the MD trajectory. CCij ranges from a value of -1, which indicates a completely anti-correlated motion between two residues, to a value of +1, which, instead, means a linearly correlated lockstep motion. In order to make the correlation matrices clear at first glimpse, we have accumulated the correlations for each SPL component, including each individual Prp8 domain and each Hsh155 HEAT-repeat, by means of correlation scores (CSs) between each SPL component and all the others. This approach, which results in a coarse and simplified variant of the CCij matrix, has been introduced to decrypt the complex correlation pattern of CRISPR-Cas9 [34,62], the intron lariat spliceosome [23], the human SF3B1 splicing factor complex [56], and the estrogen receptor alpha [55]. The result is particularly useful to capture, at first glimpse, the main dynamical trait of complex biological systems [63]. In spite of the approximation introduced in the latter, the cross-correlation matrix in its original (Figures S6–S8) and coarse form reveals a complex internal dynamics of Prp8, in which most domains move lock-step with each other, while the N-terminal one (N-term) is weakly anti-correlated with the rest of Prp8 (Figure 3A and Figures S9 and S10).

Among the RNA filaments of the intron, which should undergo a remarkable structural change to proceed toward the B* complex (i.e., the following step of the SPL cycle), negatively correlates with Prp8. This behavior is qualitatively confirmed by the different **Bact** replicas (Figure S11). Moreover, we monitored the trajectory length considered for the analysis. The last 380 ns of the production phase of the **Bact** model yielded similar results to the whole 500 ns trajectory (Figure S12). An in-depth analysis of the Hsh155 structure discloses that its cross-correlation map switches between positive and negative correlations in two regions. These are strikingly placed at H6-7 and at H14-15 (Figure 3 and Figure S6), which corresponds to the region hosting the BPA recognition site and the MDS causing mutations (the K335E and N295D). 

While the N-terminal part of the HEAT-repeats (H1-3) moves lockstep with the C-terminal (H15-20) one, the central portion (H7-14) negatively correlates with these two terminal regions. In this intricate scenario, the intron positively correlates with H1-H9 and H15-20. This confirms the result of a previous simulation study on human SF3B1 [56]. A complex communication occurs between the distinct Prp8 domains and different Hsh155 regions (Figure S13). Hence, Prp8 likely governs the motion of Hsh155, which allows it to propagate its movements toward the intron and the other SF3b proteins.

### 3.3. Molecular Framework Underlying Constitutive, Alterative, and Aberrant Splicing

**Figure 4:**
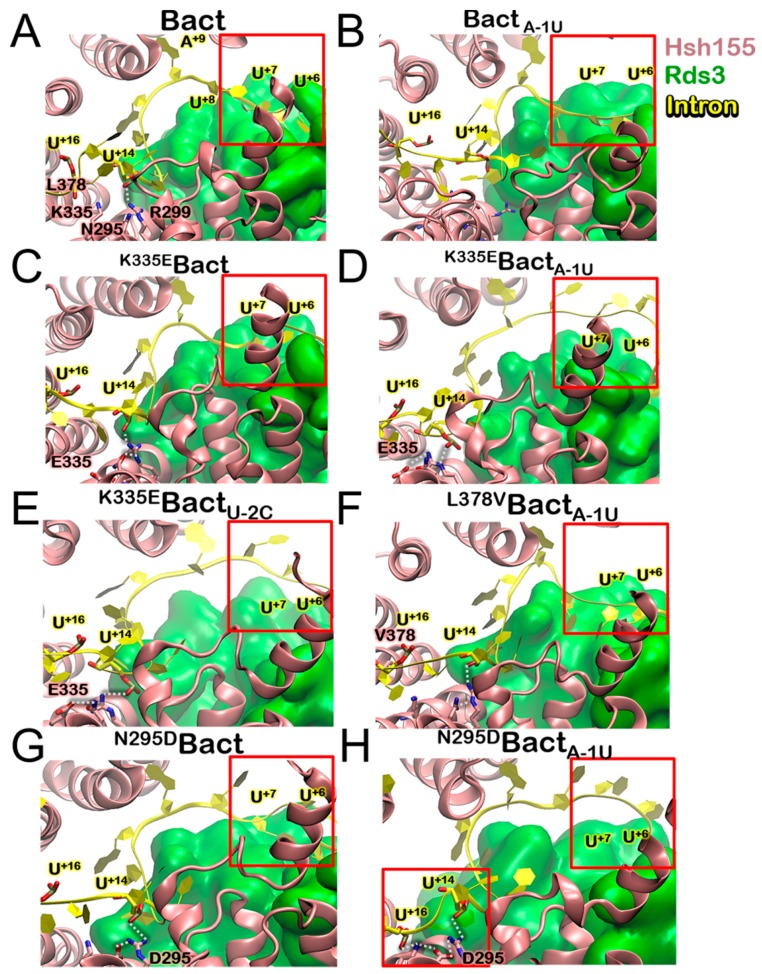
Close-up view of representative frames as extracted from the MD trajectories, depicting the intron (yellow) binding to Rds3 (green) in the nest of Hsh155 (pink) for (**A**) **Bact**, (**B**) **Bact_A-1U_**, (**C**) **^K335E^Bact**, (**D**) **^K335E^Bact_A-1U_**, (**E**) **^K335E^Bact_U-2C_**, (**F**) **^L378V^Bact_A-1U_,** (**G**) **^N295D^Bact**, and (**H**) **^N295D^Bact_A-1U_** models. Hydrogen (H)-bonds between Hsh155 residues and intron U^+14^ and U^+16^ phosphates are highlighted as white dashed lines. In the upper right corner, the red box highlights the intron/Rds3 contacts. In the lower left corner (**G**), it is depicted as the persistent H-bond network between R299, D295, K335E, and the intronic phosphates U^+14^ and U^+16^.

We next inspected how somatic mutations of Hsh155 may affect the recognition of the BPS by building seven additional 700,000 atom models: **^K335E^Bact**, **^N295D^Bact** harboring the pathogenic K335E and N295D mutations, respectively, in Hsh155, **Bact_A-1U_**, holding ncBPS (i.e., A > U mutation at the intron position -1, flanking the BPA), and **^K335E^Bact_A-1U_**, **^N295D^Bact_A-1U_**, and **^L378V^Bact_A-1U_** containing both a Hsh155 and a BPS variants. In addition, we also considered **^K335E^Bact_U-2C_** holding a distinct U-2C BPS mutation. All models were relaxed via 580 ns long MD simulations. Although the A-1U variant taken singularly induces no significant rearrangement in the H-bonds network within the BPA binding cavity, it causes a mismatch in the intron/U2 duplex, which weakens the double helix stability. Conversely, the K335E and N295D isoforms alter the inter-helical H-bond network. In the first case, the formation of a salt bridge between E335, located on H5, and R294 and R299, placed on H4 occurs. This contrasts with wild-type Hsh155 in which K335, instead, forms an intra-helix H-bond with Q338 (Figure S3). In **^N295D^Bact**, instead, an inter-helical H-bond between D295 and K335 is formed, which results in persistent contacts with the intron’s poly-pyrimidine region. In **^K335E^Bact_A-1U_, ^K335E^Bact_U-2C_**, and **^N295D^Bact_A-1U_**, no significant differences are found in the H-bonds of the BPA binding cavity nor in the intron/U2 and intron/Hsh155 interactions (Table S2). Remarkable changes, instead, occur at the intron/Rds3 interface, ranging from the BPA flanking region to that hosting the K335E and N295D mutations (Figure 4, Figure S14 and Table S2).

In **^K335^Bact_A-1U_**, **^K335^Bact_U-2C_**, and **^N295D^Bact_A-1U_**, the intron loosely binds to Rds3 and no interactions are established between U^+6^, U^+7^, and Rds3. Conversely, in **Bact**, **Bact _A-1U,_^K335^Bact, ^N295D^Bact**, and **^L378V^Bact _A-1U_**, these bases are tightly engulfed inside Rds3 thanks to the formation of persistent H-bonds with K56, N57, L63, N64, R99, N100, and E102 (Figure S14). Additionally, the H-bond network between the Rds3 and Hsh155 dwindles in **^K335^Bact_A-1_**U and **^K335^Bact_U-2C_**, while being fully persistent in **Bact**, **^K335E^Bact**, and even in **^N295D^Bact_A-1U_** (Figure S3, Table S2). As a final check, we have analyzed the impact of a non-pathogenic mutation located in the vicinity of those investigated above. Since many pathogenic mutations are mapped in this region, while non-pathogenic ones have been largely overlooked in previous studies, we annotated the latter by performing SF3B1 gene sequencing studies and an analysis of public databases. Among the mutations annotated here (Table 1), we selected the L378V variant to perform MD simulations, since this was predicted as benign with higher confidence. This mutation, simulated in the presence of the A-1U ncBPS, did not alter the Hsh155 internal dynamics nor caused a repositioning of intron (Figure 4), which is consistent with its predicted non-pathogenicity.

While no apparent electrostatic origin can be ascribed to the intron rearrangement observed at its interface with Rds3 (Figures S4 and S5), we observe that the ncBPS in **Bact_A-1U_** affects the flexibility of H5-8 (Figure S15). In addition, K335E in **^K335E^Bact** only alters H5 flexibility, while, in **^K335E^Bact_A-1U_** and **^K335E^Bact_U-2C_**, the flexibility of H3-11 slightly increases, which is consistent with the likely disentanglement of the intron from the SF3b complex suggested experimentally [7]. In **^N295D^Bact_A-1U_**, the Hsh155 flexibility increase is more modest and occurs in the presence of the cBPS, which is not relevant for intron detachment. On the opposite side, in **^L378V^Bact_A-1U_**, the Hsh155 flexibility is not altered.

### 3.4. Impact of the Bact Isoforms on Constitutive/Alternative/Aberrant Splicing

The variations of cross-correlation matrices (Figure 3 and Figures S6–S8) unveil that ncBPS binding alone does not significantly alter the communication between Prp8, Hsh155, and the distinct RNA filaments. Conversely, it affects the internal dynamics of Hsh155, where the switch regions of positive/negative correlation become less defined. When inserting only the K335E or N295D (**^K335E^Bact** and **^N295D^Bact**), the cross-correlation map becomes more similar to that of **Bact**. Strikingly, a change of Hsh155 internal correlation and dynamics occurs in the simultaneous presence of K335E and two different ncBPS sequences, which is in line with experimental evidence [7]. We find a different pattern in the intra-Hsh155 correlations with the degree of negative correlations rising. Namely, H1-11 moves oppositely to H12-20, with the only switch point of the cross-correlation map being located at H10-12. Conversely, in **^N295D^Bact_A-1U_**, a lack of correlation is visible at H11-12 (Figure S11), which assesses the pivotal role of this region for signal propagation within Hsh155 as a hallmark of HEAT-repeat proteins [20,21,64]. However, we remark that this analysis is qualitative and possibly plagued by the time scale of the simulations. Hence, it is employed to gain a coarse picture of the main alterations in the dynamical traits of domains/proteins induced by the studied mutations. As a result, the **Bact** models (even considering the distinct replicas) are all similar to non-pathological variants (with either BPS or Hsh155 single mutants or a non-pathological double mutant), while being different from the investigated pathological variants. 

**Figure 5:**
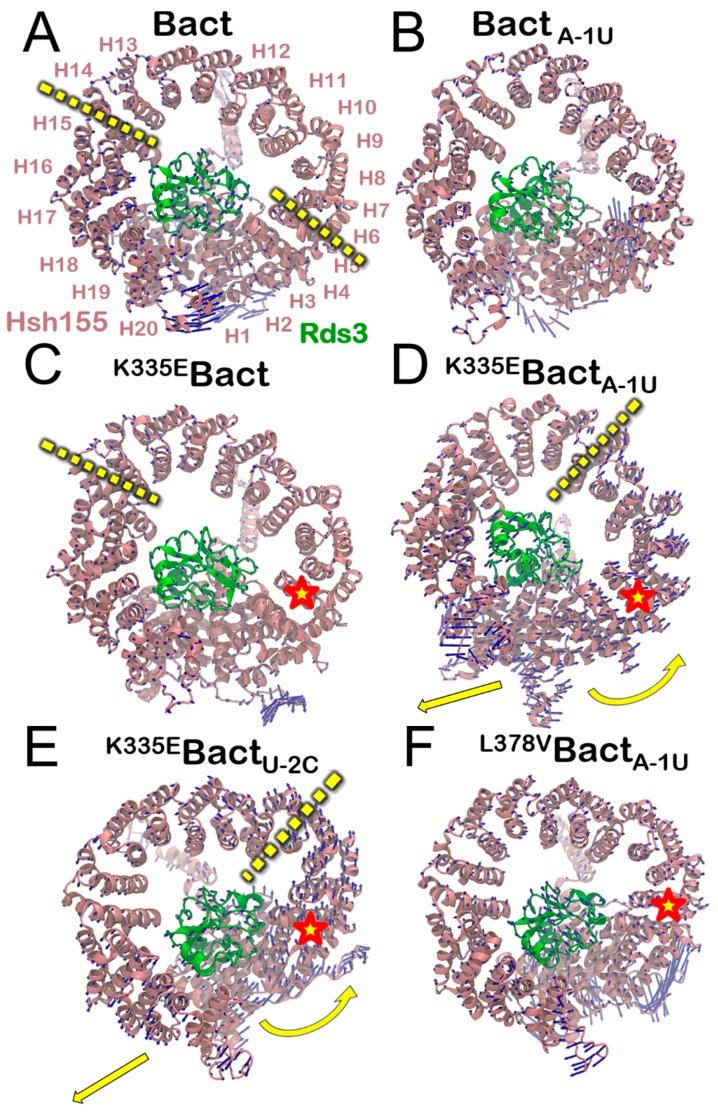
Essential dynamics as revealed by principal component analysis (PCA) for the Hsh155 protein in the (**A**) **Bact**, (**B**) **Bact_A-1U_**, (**C**) **^K335E^Bact**, and (**D**) **^K335E^Bact_A-1U_** (**E**) **^K335E^Bact_U-2C_**, and (**F**) **^L378V^Bact_A-1U_** models. (**A**) SF3b complex with labelled HEAT (huntingtin elongation factor 3 protein phosphatase 2A, target of rapamycin 1)-repeats of Hsh155 (pink), and Rds3 protein (green). The site of mutation is marked with a red star. Blue arrows show the motion of Cα atoms along the first eigenvector. Yellow dashed arrows highlight the hinges present in **Bact, ^K335E^Bact_A-1U_**, and **^K335E^Bact_U-2C_** models, and curved arrows highlight distinctive directions of motions in **^K335E^Bact_A-1U_** and **^K335E^Bact_U-2C_** models.

In order to visualize the large-scale collective motions, we performed principal component analysis (PCA), extracting the essential dynamics of the systems (i.e., the movements projected on the first PC) [52]. This analysis enables gathering valuable information on the most relevant conformational changes by taking place along MD trajectories. PCs cumulative contribution (Figure S16) shows that the contribution of the first PCs to the overall SPL motion are almost equivalent. We focused our PCA on Hsh155, as this protein exhibited most of the changes in the cross-correlation matrix detailed above. In **Bact**, the largest eigenvector’s projections are mainly located on the N-term and C-term regions of Hsh155 and are pointing in opposite directions, consistently with the anti-lockstep motion discussed previously (Figure 5, Figure S15 and Movie S1).

This analysis strikingly unveils that Hsh155 moves similarly to a “spring-pulling” in opposite directions (Movie S1), likely because of the cooperative action of the distinct Prp8 domains and the super-helical Hsh155 HEAT-repeat structure. This motion is also confirmed by X-ray and cryo-EM and MD simulation studies on human SF3B1 [20,21,56]. The observed movement is not significantly affected upon ncBPS binding, which reinforces the experimental hypothesis of the Hsh155 ability to recognize and process both consensus and non-consensus intronic sequences. The K335E mutation decreases the amplitude of the spring-pulling motion (Figure 5 and Figure S17), while, if combined with two different ncBPSs, it magnifies it (Movie S2). Conversely, N295D does not significantly alter the Hsh155 internal dynamics neither in the presence of cBPS nor ncBPS. However, we remark that the structural rearrangement occurring at the mutation site affects intron binding to Rds3 only in the presence of ncBPSs (Figure S14). Stunningly, this is a common structural trait of all pathogenic cases investigated in this scenario. Consistently with experimental evidences [7], an intron dis-engagement may be associated with a facilitated release of ncBPS, which results in a possible translation/recognition of the SPL toward/of a different (likely cryptic and erroneous) 3’SS, which may adversely affect splicing.

## 4. Discussion

The SPL B^act^ complex has been recently a subject of major structural and functional breakthrough due to a significant number of cryo-EM maps trapping the yeast [18] and the human homologues [2,19] in distinct conformational states. This study focuses on the yeast B^act^ from *S. cerevisiae* since this was the first B^act^ structure released providing key structural insights on intron recognition. The B^act^ complex assembles before the first splicing step. As such, the system investigated in this case is almost primed for catalysis.

Remarkably, the cumulative multi-µs MD simulations of the **Bact** model preserves the recognition and active sites, in line with their tight stability across the splicing cycle documented by distinct cryo-EM structures and by our previous simulations of intron lariat system (ILS) from *Schizosaccharomyces pombe* [23]. In **Bact**, the BPS is engaged in a long duplex with U2 snRNA, and lays at a large distance from the scissile G-G bond of the 5’SS. In fact, SPL, to move toward the next step of the cycle, has to undergo a major conformational change in order to bring the BPA and the 5’SS in close proximity, which enables the occurrence of the first splicing step. These events are most likely mediated by Prp8, and in particular by its RNase-H domain, which in B^act^ adopts a distinct conformation from the other states trapped by cryo-EM [65]. In our previous study of ILS, the RNase-H domain negatively correlated with the rest of Spp42 (Prp8 in *Saccharomyces cerevisiae*), whereas in the **Bact** model studied, a predominantly lockstep motion of the RNase-H domain with the rest of Prp8 is recorded. The B^act^ structure investigated here contains the Hsh155, a protein of the SF3b factor, which is responsible for the recognition of BPA, the flanking intronic sequence, and in the selection of the correct 3’SS. Frequent Hsh155 mutations are associated with an altered gene expression and the onset of SPL-mutant cancers, with a most-likely conserved mechanism between humans and yeast [7,66,67].

In spite of the pivotal role of Hsh155/SF3B1 in intron recognition and in the onset of SPL–related pathologies, the structural and dynamic impact of its point mutations on intron selection remains obscure. Hence, this study is mainly devoted to assess the functional role of Hsh155. This protein has a peculiar super-helical structure formed by 20 HEAT-repeats, with each composed of two anti-parallel α-helices. Its essential dynamics shows that Hsh155 undergoes a functional spring-pulling-like movement, occurring via a twist of the protein at two hinge points located between H5-H6 and H14-15 (Figure 3).

Consistently with experimental findings, our simulations disclose that Hsh155 is able to bind even ncBPS sequences, possibly modulating in this manner the splicing of distinct pre-mRNAs (alternative splicing). All nucleotides forming the BPS, with the only exception of the BPA (A1), are recognized by Hsh155 via H-bonds to the phosphate backbone only, which decreases the specificity of BPS selection and enabling, as a result, alternative splicing. By introducing a transversion mutation (A-1U or U-2C) immediately downstream of the BPA, a mismatch is generated. Our simulations reveal that these mutations weaken the intron/U2 duplex, while only slightly affecting the overall arrangement of the intron, and inducing a minimal perturbation of the correlated motions.

On the other hand, we show that MDS mutations (here K335E or N295D) remarkably alter intron binding when containing ncBPSs, without affecting that containing cBPSs. The essential dynamics of the **^K335E^Bact** in the presence of two distinct ncBPS (A-1U and U-2C) shoots an enhanced spring-pulling-like motion of Hsh155, while the cross-correlation matrix pinpoints a change of the internal dynamics achieved thanks to the segmentation of HEAT-repeats into two major regions (Figure 3, Figures S6–S8). Although less markedly altering the Hsh155 internal dynamics, a second pathogenic N295D variant, in the presence of the A-1U ncBPS, also destabilizes intron binding to Rds3. The critical importance of the Hsh155 HEAT-repeats is further corroborated by our exploration of non-pathogenic Hsh155 isoforms near the H5 region. Our functional annotation (Table 1) reveals that few and rare non-pathogenic single nucleotide polymorphisms are present in this region of Hsh155, and that, among these, the L378V does not impact the functional dynamics nor intron recognition even when Hsh155 binds ncBPS. Hence, our study reinforces the experimental hypothesis suggesting that the functional dynamics of Hsh155/SF3B1 may be a key modulator of BPS usage. A detailed inspection of MD trajectories elucidated substantial positional rearrangements of the intron due to weaker interactions with Rds3 (Figure 4). As a result, the K335E/N295D carcinogenic variants are unable to efficiently bind an intron/U2 duplex, which contains ncBPS. In this intricate scenario, Hsh155/SF3B1 acts like an accordion instrument and its HEAT-repeats bellow, which, being flawless, can produce desired sounds (the appropriate functional mRNA). The K335E/N295D mutations alters the accordion-like motion of Hsh155, impacting, as a result, on ncBPS recognition/selection.

## 5. Conclusions

Multi-µs-long MD simulations of eight distinct models of the Bact SPL complex hosting pathogenic and non-pathogenic Hsh155 variants, along with the cBPS and ncBPS sequence, and supported by gene sequencing studies, enlighten from an atomic-level perspective of the impact of Hsh155 MDS causing isoforms on the structural and dynamical properties of the Bact complex. Our study represents an unprecedented attempt to characterize the molecular principles underlying the subtle regulation of constitutive, alternative, and aberrant splicing, which contributes to a fundamental advance in the mechanistic understanding of this pivotal step of gene expression and regulation. An in-depth comprehension of splicing regulation not only discloses fundamental biological principles, but also offers appealing opportunities to devise innovative therapeutic solutions for tackling cancer and other major human diseases.

## Figures and Tables

**Table 1:** SF3B1 (Hsh155 in yeast) isoforms with unclear clinical annotation. Prediction of their effect is estimated on the basis of bioinformatics analysis (see Materials and Methods section). In brackets are the residues corresponding to yeast Hsh155.

Mutation	Associated	Prediction	Type	Annotation	Frequency
Q698E (H347)	K666E	4/6 damaging	Somatic	uncertain	<1%
Q670H (Q339)		9/9 damaging	Germline	uncertain	1%
I709V (L378)		4/9 damaging	Germline	benign	<1%
T434P (V103)		8/9 damaging	Germline	uncertain	<1%
I360V (L29)		2/9 damaging	Germline	benign	<1%

## Supplementary Materials

The following are available online at https://www.mdpi.com/2218-273X/9/10/633/s1. Figure S1: Convergence of the simulations; Figure S2: Stability of the triple helix; Figure S3: Hydrogen bond network at recognition and mutation sites; Figures S4 and S5: Electrostatic potential; Figure S6: Effect of ionic physiological strength; Figures S7 and S8: Cross-correlation matrices; Figures S9–S11: Coarse cross-correlation matrices; Figure S12: Monitoring convergence of cross correlation matrices; Figure S13: Essential dynamics of Bact; Figure S14: Intron binding to Rds3; Figure S15: Flexibility of Hsh155; Figure S16: Principal components (PCs) cumulative contribution to variance; Figure S17: Essential dynamics of ^N295D^Bact, and ^N295D^Bact_A-1U_. Table S1: Details of Bact model; Table S2: Hydrogen-bond analysis, and Movies S1 and S2.
