# Supplementary material for: Disclosing the Impact of Carcinogenic SF3b Mutations on Pre-mRNA Recognition Via All-Atom Simulations
Source: Biomolecules. 2019 Oct 21;9(10):633. doi: 10.3390/biom9100633 (PMC6843770; doi:10.3390/biom9100633)
Supplement: Supplementary file 1 [file biomolecules-09-00633-s001.pdf]

Supplementary Information

Disclosing the impact of carcinogenic SF3b mutations on pre-mRNA recognition via all-atom simulations

Jure Borišek[1,2], Andrea Saltalamacchia[3], Anna Galli[4], Giulia Palermo[5], Elisabetta Molteni[4], Luca Malcovati[4], Alessandra Magistrato[1*]

[1]CNR-IOM-Democritos National Simulation Center c/o SISSA

[2]National Institute of Chemistry, Slovenia

[3]International School for Advanced Studies (SISSA)

[4]Department of Molecular Medicine, University of Pavia & Department of Hematology, IRCCS S. Matteo Hospital Foundation

[5]Department of Bioengineering, University of California Riverside, Riverside CA 92521, United States

Corresponding Author*:

*E-mail: alessandra.magistrato@sissa.it

Table of contents

Supplementary Figures S3
Figure S1........ S3
Figure S2........ S4
Figure S3........ S5
Figure S4........ S6
Figure S5........ S8
Figure S6........ S10
Figure S7........ S11
Figure S8........ S13
Figure S9........ S14
Figure S10........ S16
Figure S11........ S17
Figure S12........ S19
Figure S13........ S20
Figure S14........ S21
Figure S15........ S23
Figure S16........ S24
Figure S17........ S25
Supplementary Tables S26
Table S1. .... S26
Table S2. .... S27
Supplementary Movies S28
Movie S1. .... S28
Movie S2. .... S29

Supplementary Figures

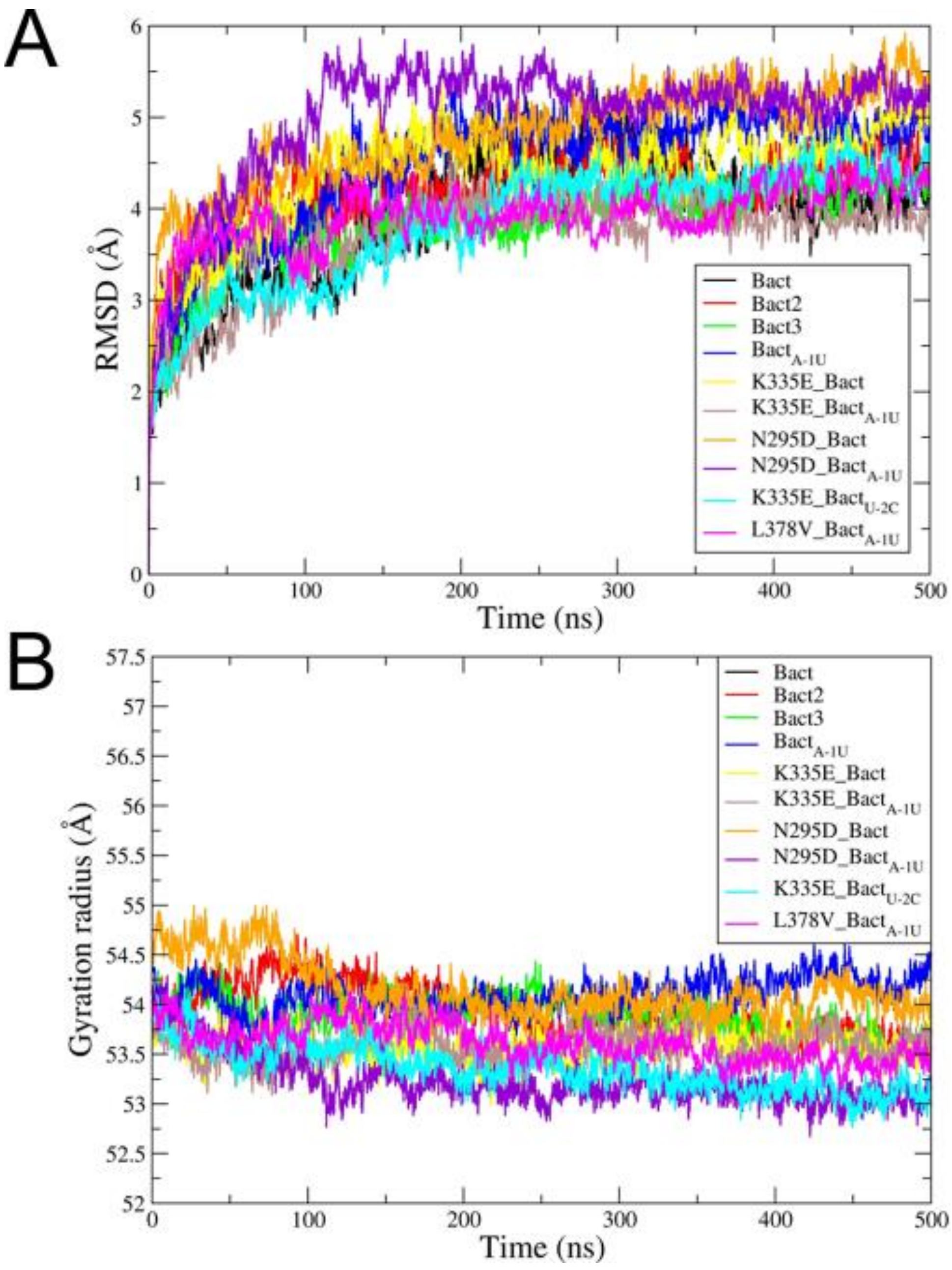

Figure S1. Convergence of the simulations. (A) Root Mean Square Deviations (RMSD)s and (B) Radius of gyration (Rg) vs. simulation time (ns) calculated on the production phase of Molecular Dynamics (MD) trajectories for models: **Bact**, **Bact**$_{\mathbf{A-1U}}$, $^{\mathbf{K335E}}$**Bact**, $^{\mathbf{K335E}}$**Bact**$_{\mathbf{A-1U}}$, $^{\mathbf{K335E}}$**Bact**$_{\mathbf{U-2C}}$, $^{\mathbf{L378V}}$**Bact**$_{\mathbf{A-1U}}$, $^{\mathbf{N295D}}$**Bact,** $^{\mathbf{N295D}}$**Bact**$_{\mathbf{A-1U}}$, **Bact2** and **Bact3**. The latter two refer to replicas of the Bact model. The other Bact models are labeled reporting the Hsh155 and BPS mutation as left superscript and right subscript, respectively.

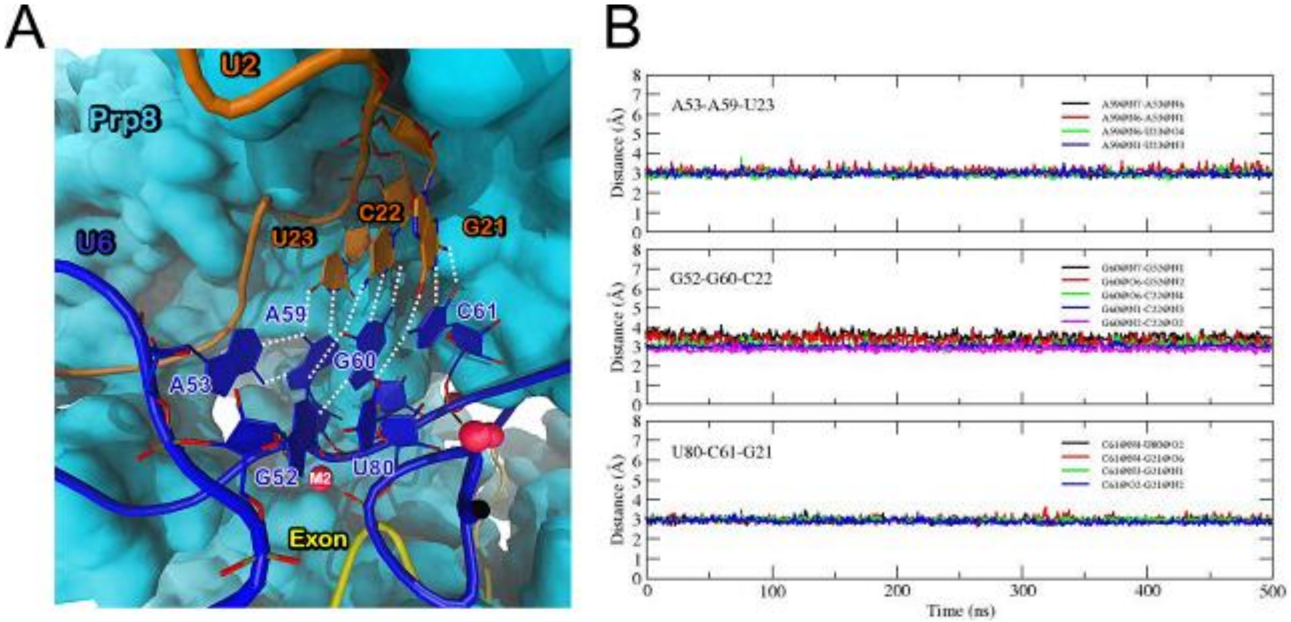

Figure S2. Stability of the triple helix. (A) Representative snapshot of the triple-helix motif nested in the catalytic site of the **Bact** model as extracted from the last 500 ns of MD simulations. U2 and U6 snRNAs are represented as orange and blue tubes, respectively. Mg^{2+} ions are depicted as light red spheres with the catalytic Mg^{2+} ion labeled as M2. The nucleotides involved in the triple-helix are pictured in licorice. The Prp8 protein is shown in cyan surface. Hydrogen bonds (H-bonds) between RNA base-pairs are depicted as white dashed lines. (B) Time evolution (ns) of the H-bonds distances (Å) between base-pairs of the nucleotides involved in the triple-helix: A53-A59-U23, G52-G60-C22 and U80-C61-G21 for wild-type **Bact** model.

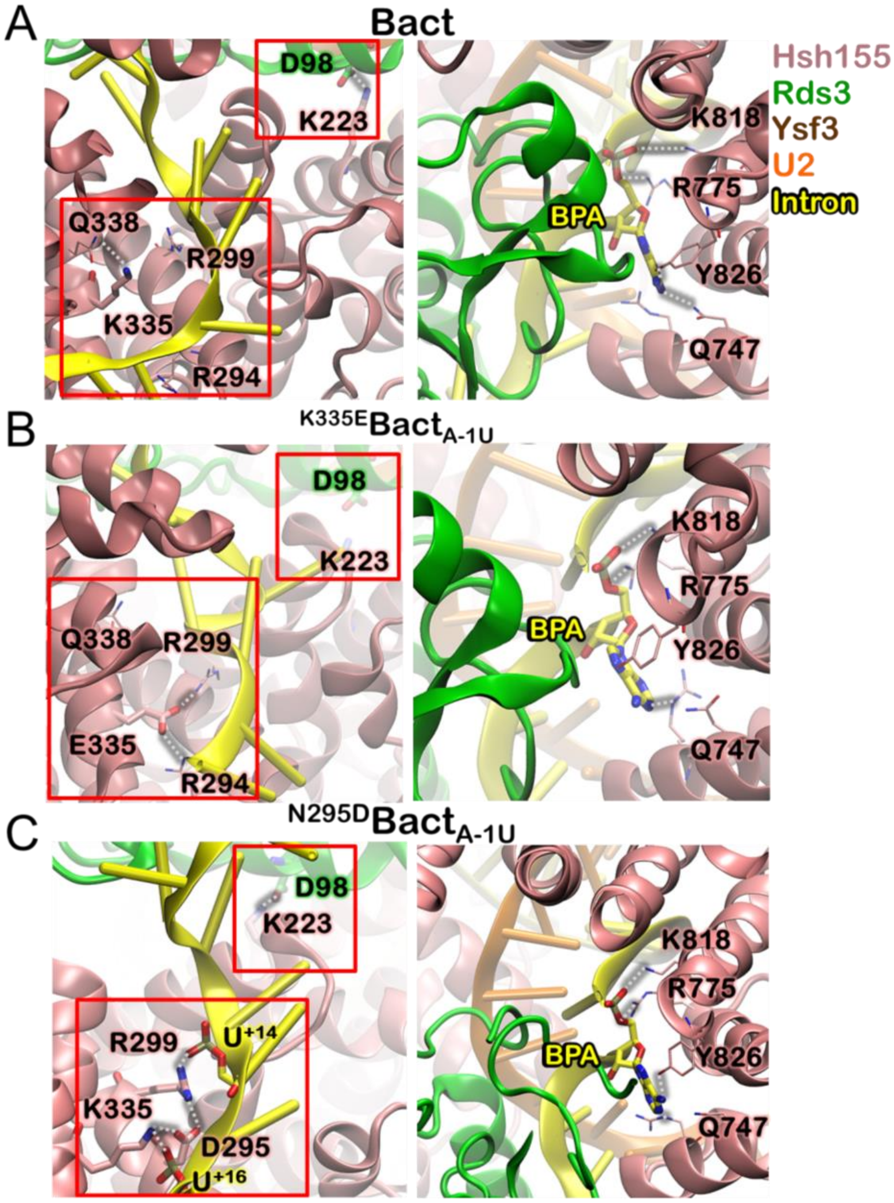

Figure S3 Hydrogen bond network at recognition and mutation sites. Representative snapshots of the hydrogen-bond network within the K335E and N295D mutation site and the Hsh155-Rds3 interaction interface (left panel) and of the branch point adenosine (BPA) binding site (right panel) for (A) **Bact**, (B) $^{\mathbf{K335E}}\mathbf{Bact}_{\mathbf{A\text{-}1U}}$ and (C) $^{\mathbf{N295D}}\mathbf{Bact}_{\mathbf{A\text{-}1U}}$ models as extracted from the last 500 ns of MD simulations. Hsh155, Rds3, U2 and intron are depicted as pink, green, orange and yellow new cartoons, respectively. The BPA is shown in licorice with C atoms colored in yellow.

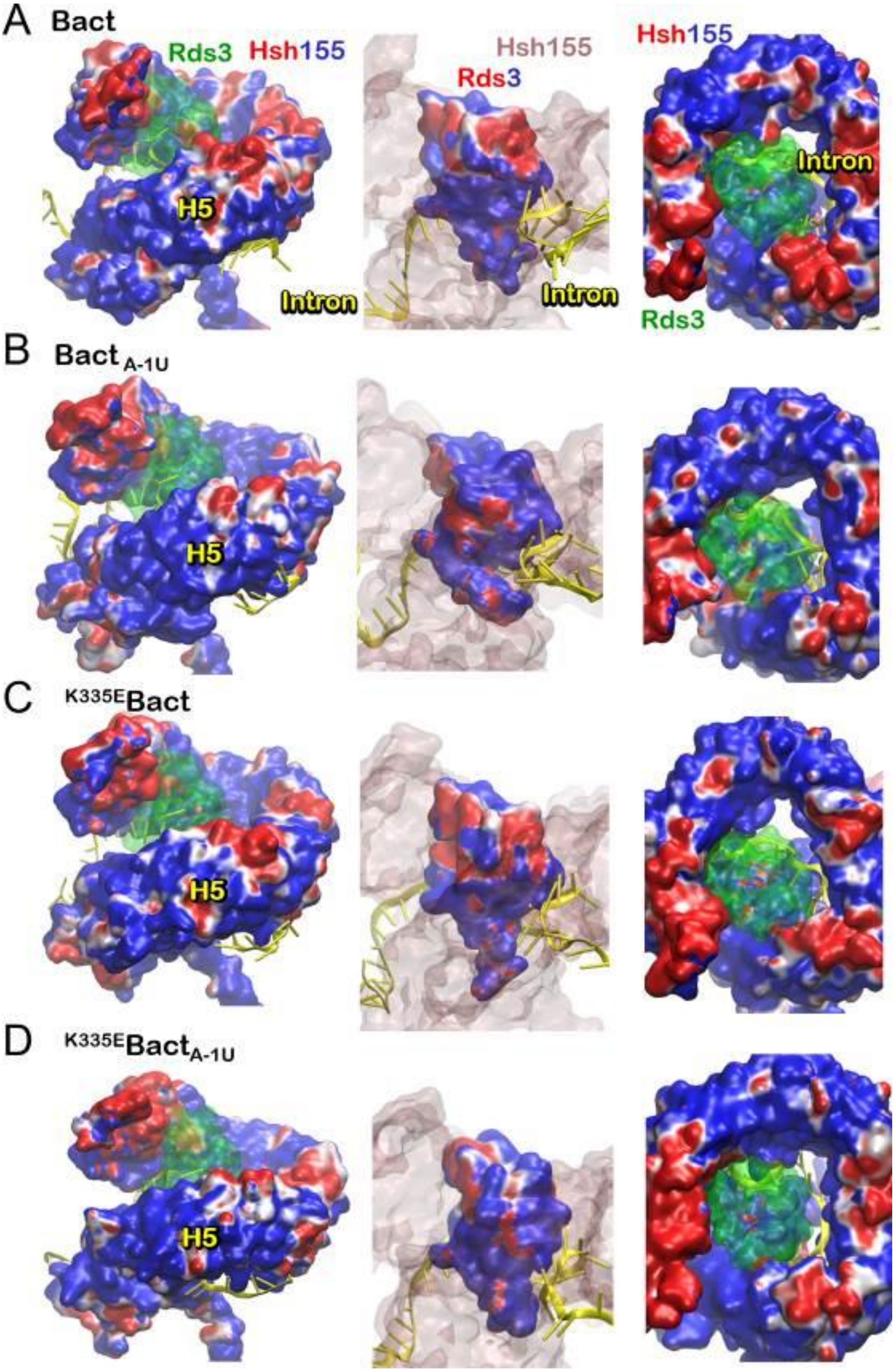

Figure S4. Electrostatic potential. Representative structures as extracted from a cluster analysis of the last 500 ns of MD trajectories. The electrostatic potential, calculated with

APBS program, at the interface between Hsh155 and Rds3 proteins and intron is shown from different perspectives for (A) **Bact**, (B) **Bact$_{A\text{-}1U}$**, (C) **^{K335E}Bact**, and (D) **^{K335E}Bact$_{A\text{-}1U}$** systems. The first column depicts the electrostatic potential of Hsh155, while Rds3 is shown in green transparent surface and intron in yellow new cartoon. The mutation site of Hsh155 (labeled as H5 HEAT-repeat domain and depicted in yellow) is highlighted. In the middle panel, the electrostatic potential of Rds3 is shown and Hsh155 is represented as transparent pink surface to show the interacting surface between the two proteins. In the right panel, the same representation of the first column is shown from a top view. In all Figure the positive and the negative potential are displayed as blue and red surfaces, respectively. In **^{K335E}Bact** the presence of E335 slightly induces a decrease in the positive potential on Hsh155 external surface, while no relevant change takes place in the positive electrostatic potential of the channel trapping the intron at the Rds3/Hsh155 interface. The same holds true for **Bact$_{A\text{-}1U}$** and **^{K335E}Bact$_{A\text{-}1U}$** models where the slight rearrangement of the intronic bases flanking the A-1U mutation increases the negative potential of the Rds3/Hsh155 interacting surface. The outer electrostatic surface of Hsh155 H5 region surrounding the mutation site is similar in all models.

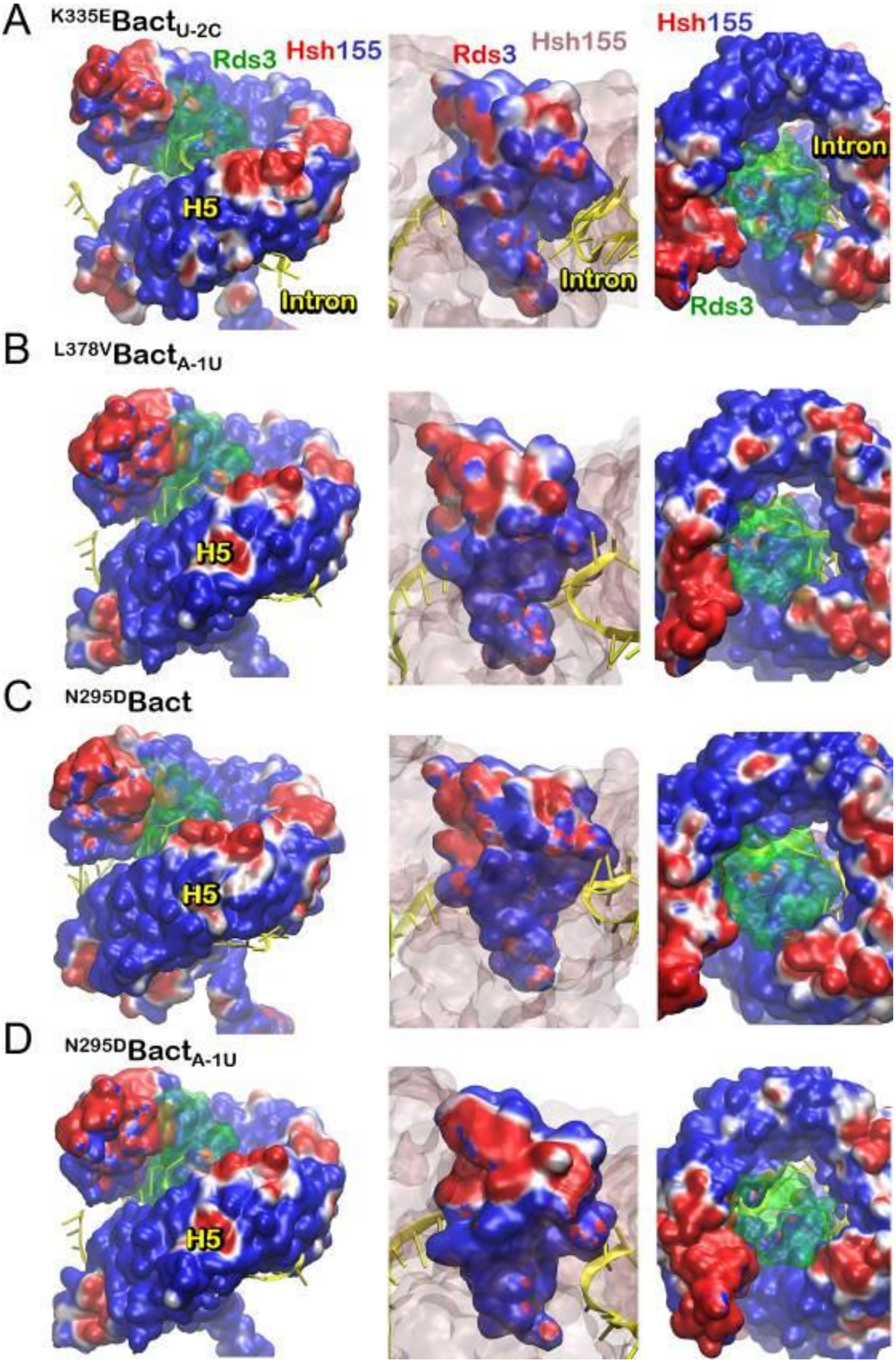

Figure S5. Electrostatic potential. Representative structures as extracted from a cluster analysis of the last 500 ns of MD trajectories. The electrostatic potential, calculated with APBS program, at the interface between Hsh155 and Rds3 proteins and intron is shown

from different perspectives for (A) ^{K335E}**Bact$_{U-2C}$**, (B) ^{L378V}**Bact$_{A-1U}$**, (C) ^{N295D}**Bact**, and (D) ^{N295D}**Bact$_{A-1U}$** systems. The first column depicts the electrostatic potential of Hsh155, while Rds3 is shown in green transparent surface and intron in yellow new cartoon. The mutation site of Hsh155 (labeled as H5 HEAT-repeat domain and depicted in yellow) is highlighted. In the middle panel, the electrostatic potential of Rds3 is shown and Hsh155 is represented as transparent pink surface to show the interacting surface between the two proteins. In the right panel, the same representation of the first column is shown from a top view. In all Figure, the positive and the negative potential are displayed as blue and red surfaces, respectively.

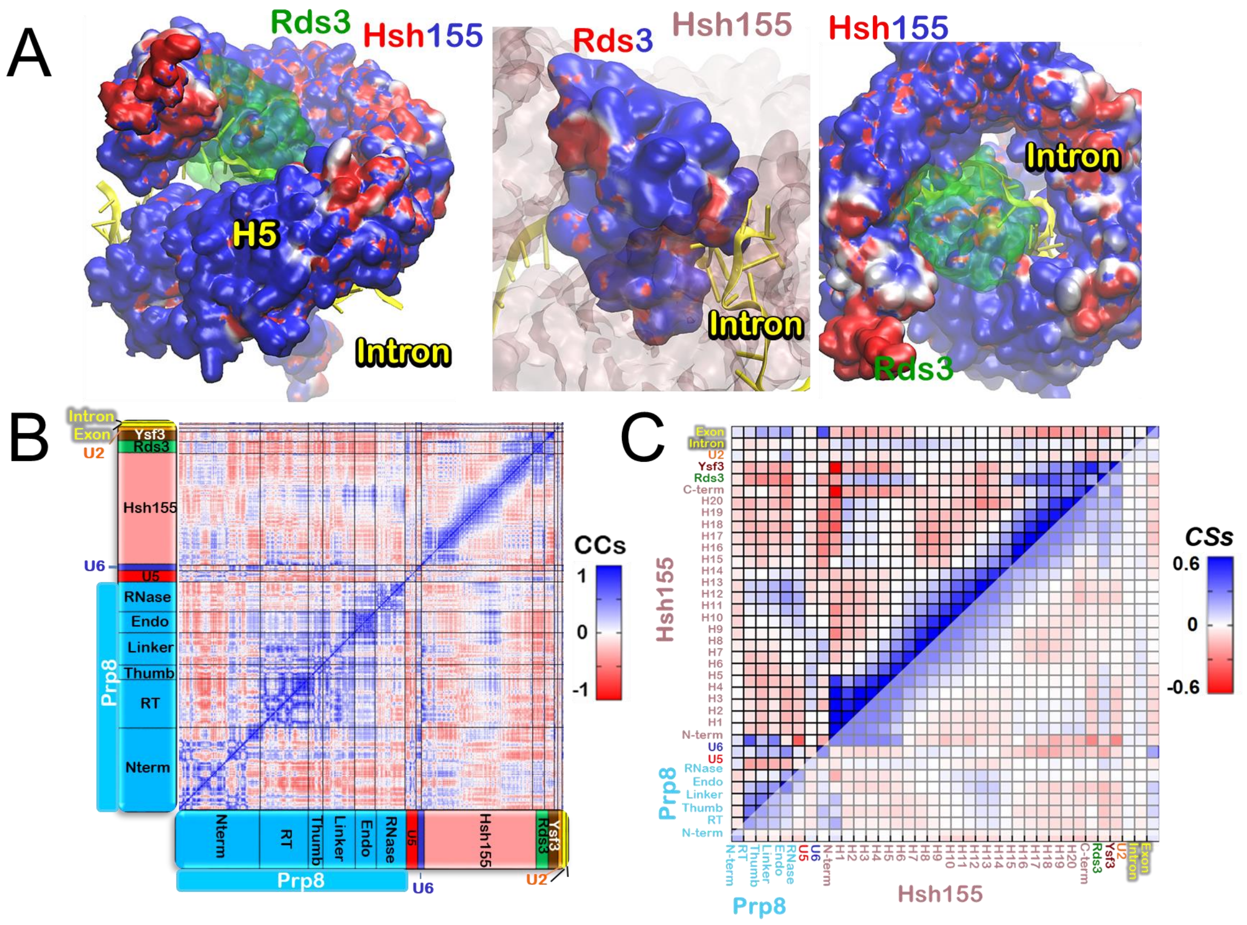

Figure S6. Effect of ionic physiological strength. Results of a control MD simulation of **Bact** model in presence of 0.15 M KCl salt concentration. (A) The electrostatic potential, calculated with APBS program, at the interface between Hsh155 and Rds3 proteins and intron is shown from different perspectives. (B) Per-residue Pearsons' cross coefficients (CCs) cross-correlation matrix derived from the mass-weighted covariance matrix ranging from -1 (red, anti-correlated motions) to +1 (blue, correlated motions). (C) A density correlation scores (CSs) reported in the range from -0.6 to 0.6 for clarity reasons. The Hsh155 protein is split by HEAT-repeat domains. Each column and row reports the sum of CSs values calculated between a specific component and the rest.

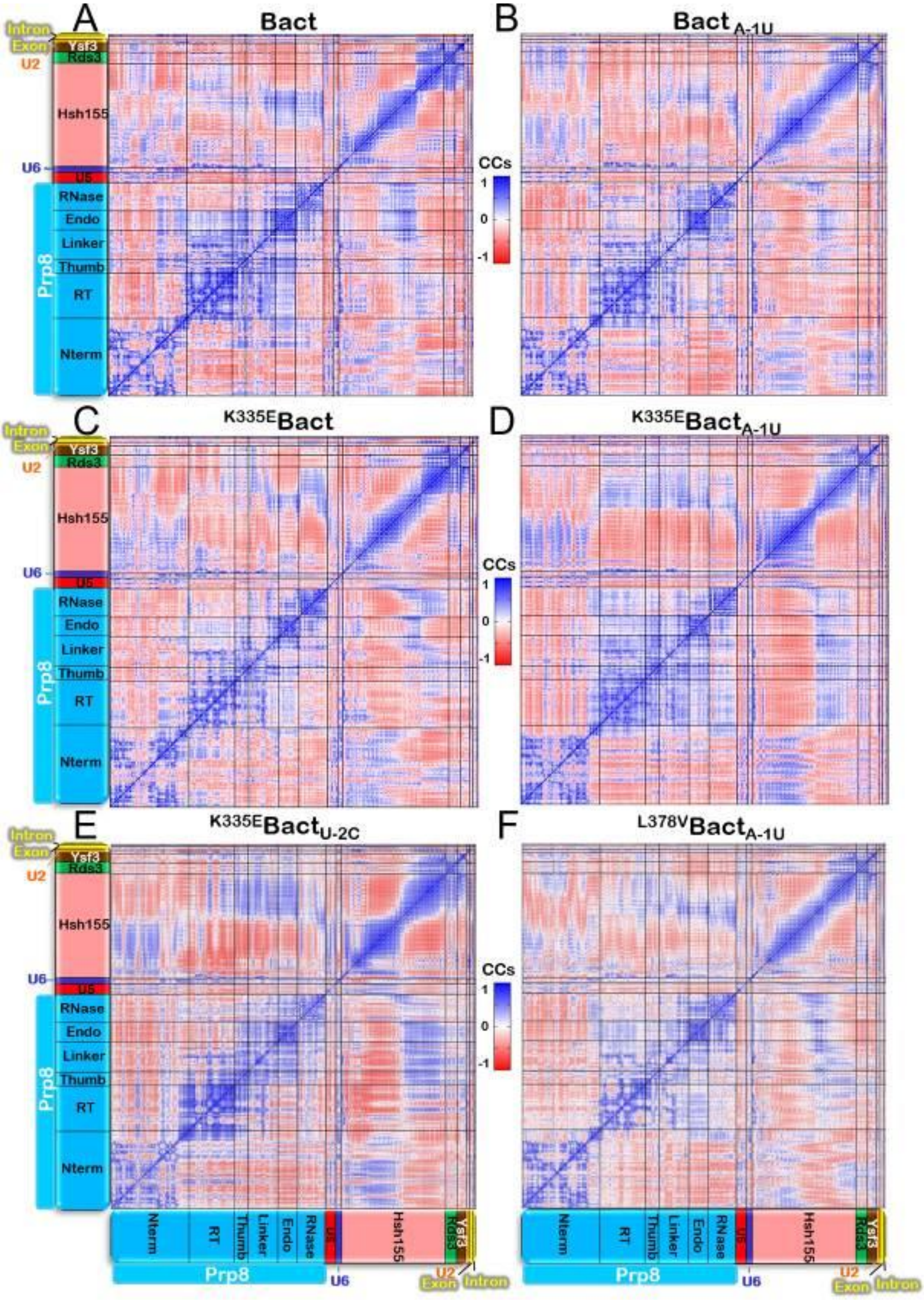

Figure S7. Cross-correlation matrices. Cooperative motion leading the functional

dynamics of the Bact models investigated. Per-residue Pearsons' cross coefficients (CCs) cross-correlation matrix derived from the mass-weighted covariance matrix calculated over the last 500 ns of MD trajectories. CCs values range from -1 (red, anti-correlated motions) to +1 (blue, correlated motions). Depicted are CCs for (A) **Bact**, (B) **Bact$_{A\text{-}1U}$**, (C) **^{K335E}Bact**, (D) **^{K335E}Bact$_{A\text{-}1U}$**, (E) **^{K335E}Bact$_{U\text{-}2C}$**, (F) **^{L378V}Bact$_{A\text{-}1U}$** models. The protein names and their domains are reported on the bottom and on the left side, highlighted with boxes of different colors.

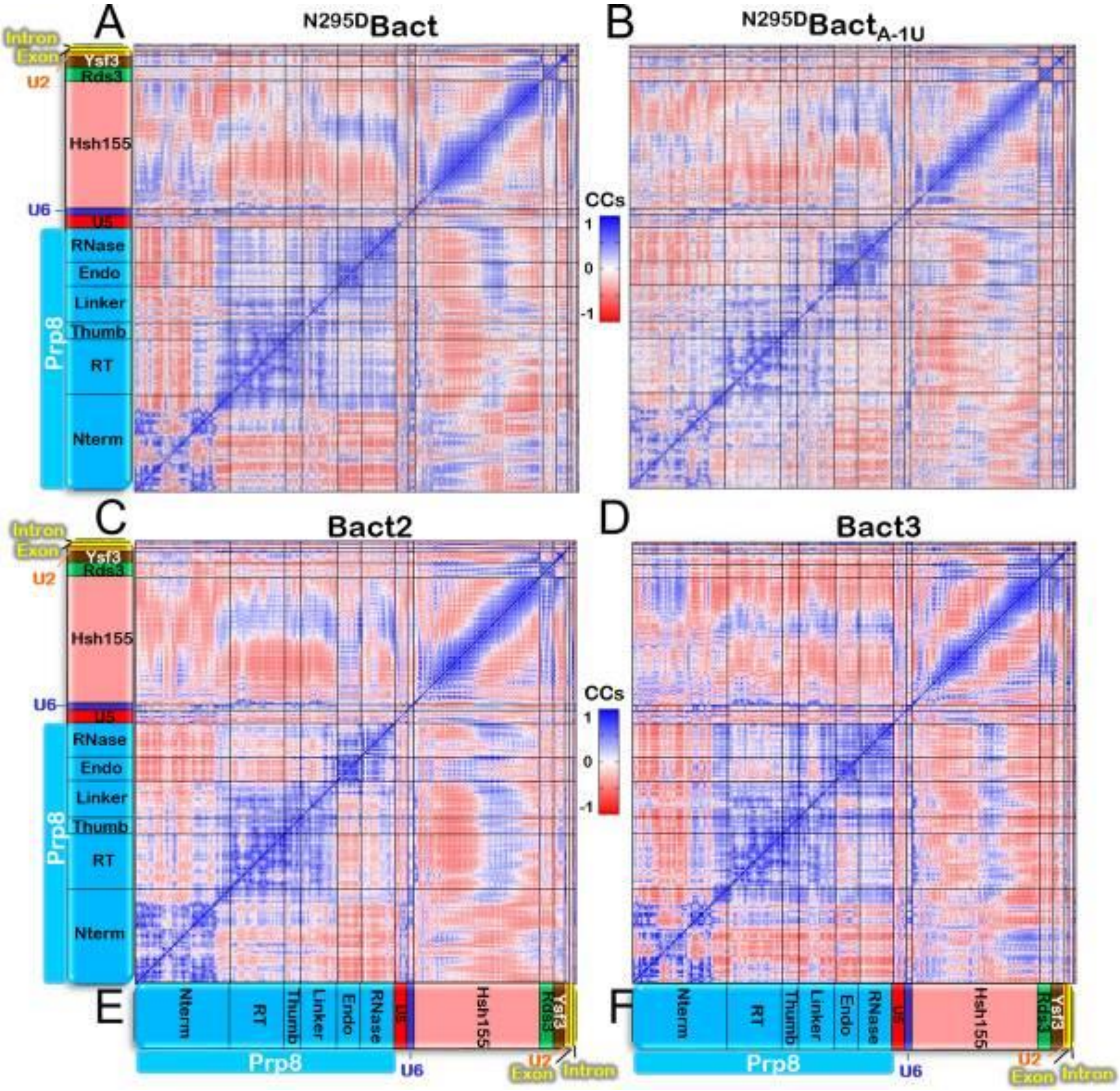

Figure S8. Cross-correlation matrices. Cooperative motion leading the functional dynamics of the Bact models investigated. Per-residue Pearsons' cross coefficients (CCs) cross-correlation matrix derived from the mass-weighted covariance matrix calculated over the last 500 ns of MD trajectories. CCs values range from -1 (red, anti-correlated motions) to +1 (blue, correlated motions). Depicted are CCs for (A) ^{N295D}**Bact** and (B) ^{N295D}**Bact$_{A-1U}$** models and **Bact** replicas (C) **Bact2** and (D) **Bact3**. The protein names and their domains are reported on the bottom and on the left side, highlighted with boxes of different colors.

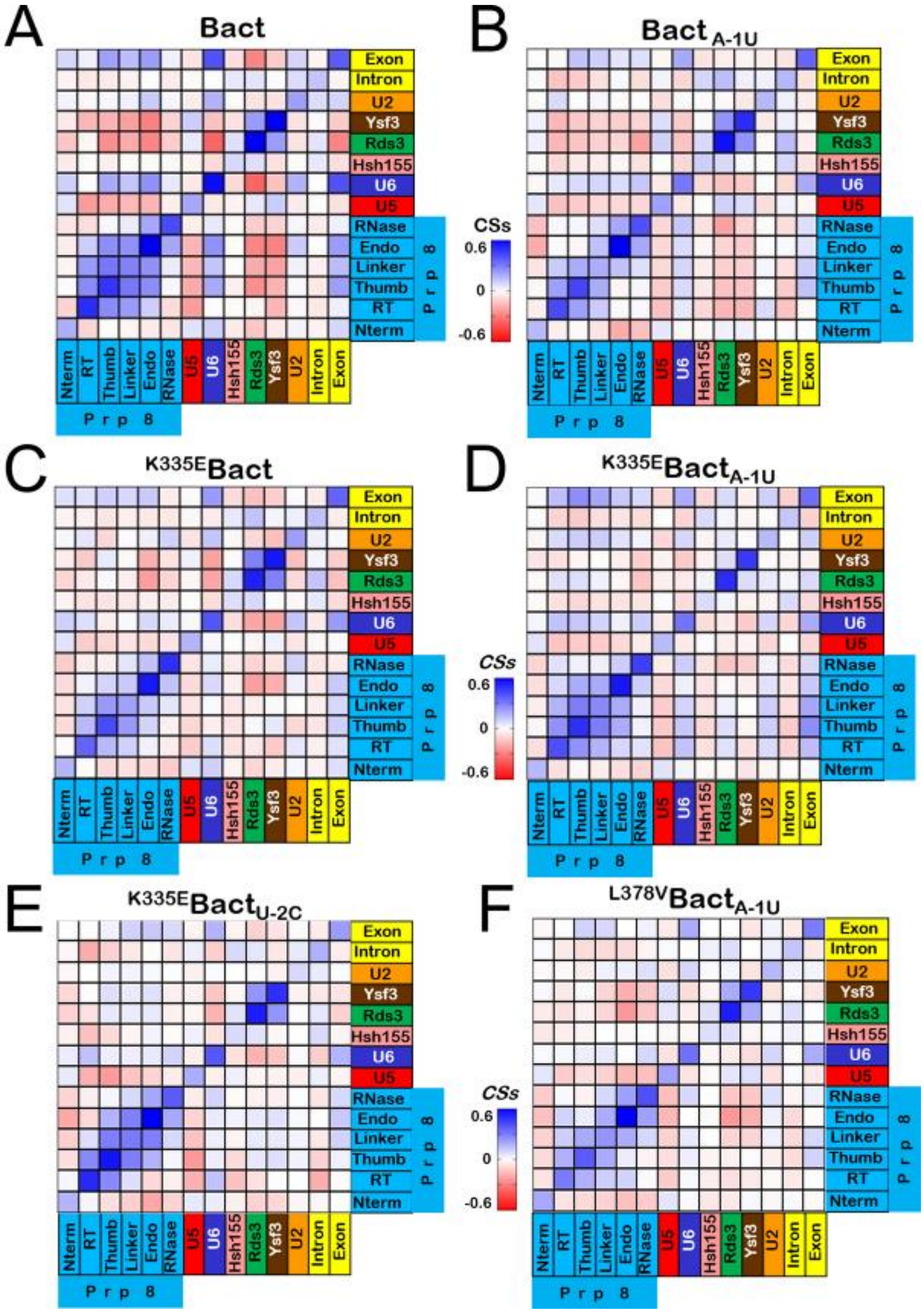

Figure S9. Coarse cross-correlation matrices. Cooperative motion underlying the functional dynamics of the B^{act} models investigated here. Per-residue Pearson's

coefficients (CCs) cross-correlation matrix is derived from the mass-weighted covariance matrix calculated over the last 500 ns of MD trajectories. CCs values range from -1 (red, anti-correlated motions) to +1 (blue, correlated motions) are summed for each pair of SPL proteins/domains, and normalized, as detailed in the Methods section of the main text, to provide a density correlation scores (CSs). CSs are shown in the range from -0.6 to 0.6 for clarity reasons. Depicted are CSs for (A) **Bact**, (B) **$\mathbf{Bact_{A\text{-}1U}}$**, (C) **$\mathbf{^{K335E}Bact}$**, (D) **$\mathbf{^{K335E}Bact_{A\text{-}1U}}$**, (E) **$\mathbf{^{K335E}Bact_{U\text{-}2C}}$**, and (F) **$\mathbf{^{L378V}Bact_{A\text{-}1U}}$** models. The names of the proteins and their domains are reported on the bottom and on the right side of each matrix, and are highlighted with boxes of different colors.

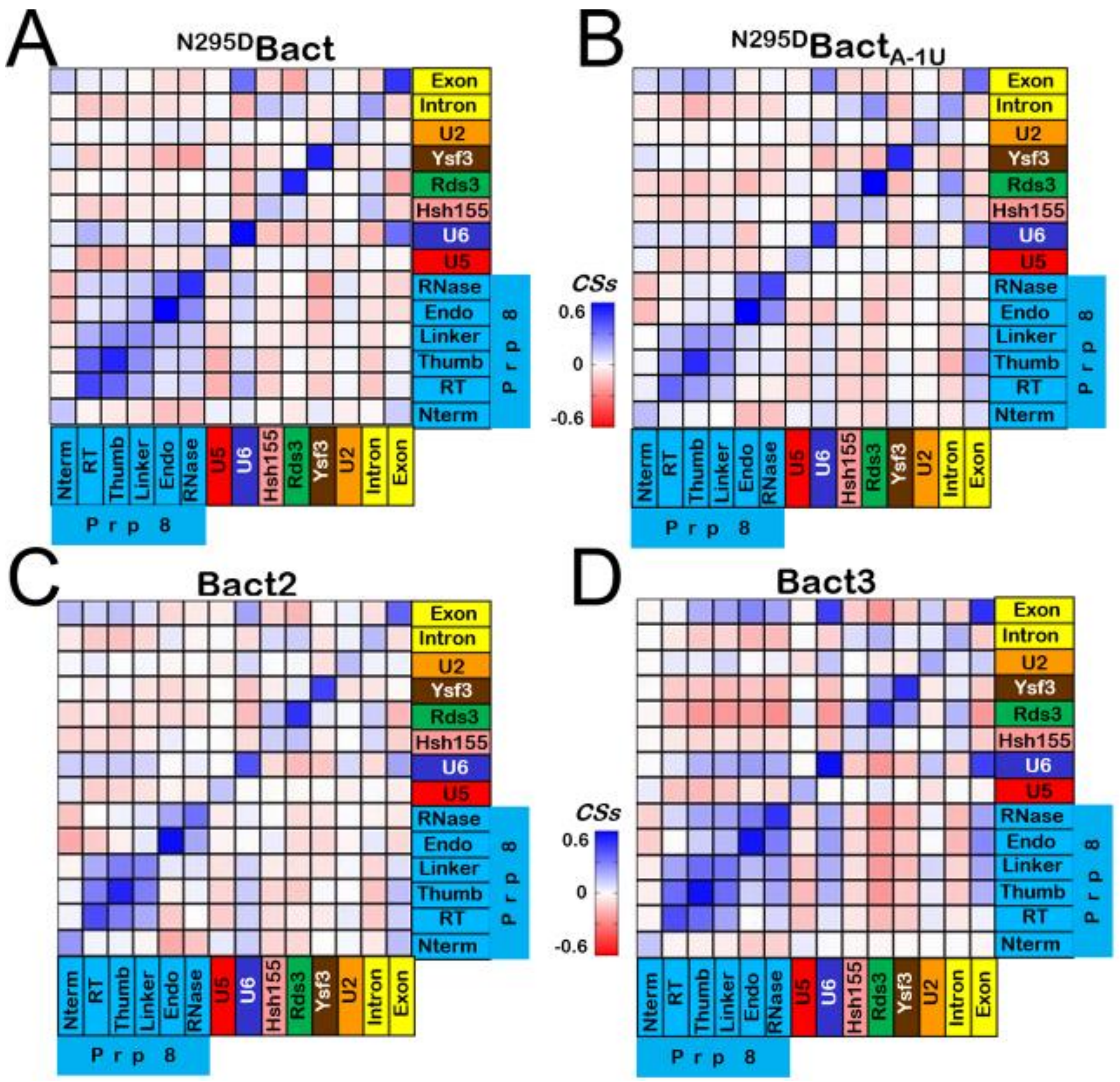

Figure S10. Coarse cross-correlation matrices. Cooperative motion underlying the functional dynamics of the B^{act} models investigated here. Per-residue Pearson's coefficients (CCs) cross-correlation matrix is derived from the mass-weighted covariance matrix calculated over the last 500 ns of MD trajectories. CCs values range from -1 (red, anti-correlated motions) to +1 (blue, correlated motions) are summed for each pair of SPL proteins/domains, and normalized, as detailed in the Methods section of the main text, to provide a density correlation scores (CSs). CSs are shown in the range from -0.6 to 0.6 for clarity reasons. Depicted are CSs for (A) ^{N295D}**Bact** and (B) ^{N295D}**Bact$_{A-1U}$** models and **Bact** replicas (C) **Bact2** and (D) **Bact3**. The names of the proteins and their domains are reported on the bottom and on the right side of each matrix, and are highlighted with boxes of different colors.

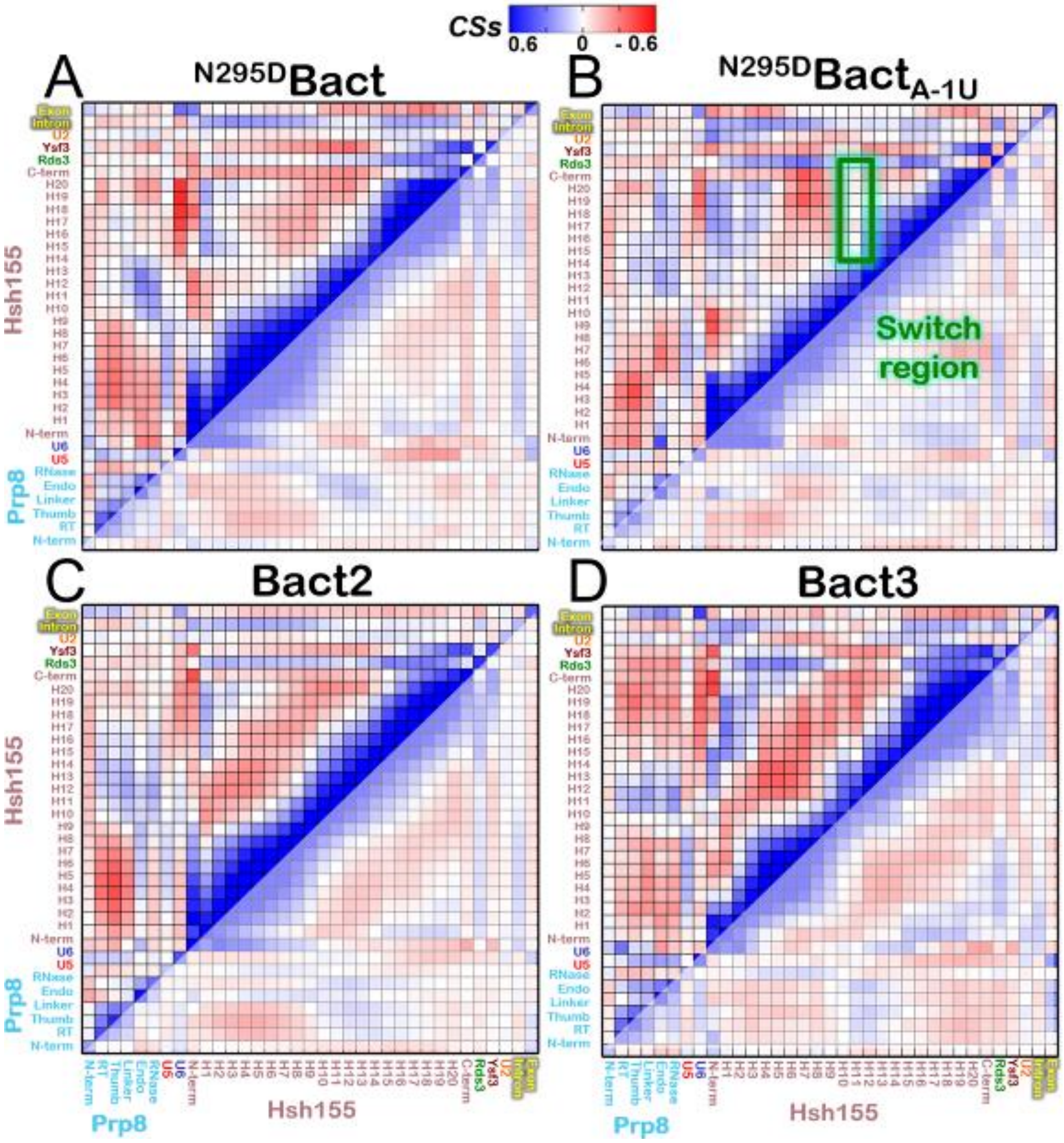

Figure S11. Coarse cross-correlation matrices. Cooperative motion leading the functional dynamics of the distinct Bact models investigated. Per-residue Pearson's coefficients (CCs) cross-correlation matrix is derived from the mass-weighted covariance matrix calculated over the last 500 ns of classical MD trajectories. CCs values range from -1 (red, anti-correlated motions) to +1 (blue, correlated motions) are summed for each pair of SPL proteins/domains, and normalized, as detailed in the Methods section of the main text, to provide a density correlation scores (CSs). CSs are here reported in the range from -0.6 to 0.6 for clarity reasons. The Hsh155 protein is here split by HEAT-repeat domains. In green

are encircled regions of Hsh155 protein in which a switch between positive and negative correlation occurs. Each column and row reports the sum of CSs values calculated between a specific component and the rest. Depicted are CSs (A) $^{\mathbf{N295D}}$**Bact** and (B) $^{\mathbf{N295D}}\mathbf{Bact_{A\text{-}1U}}$ models and (C) **Bact2** and (D) **Bact3**, which are replicas of the **Bact** model. The protein name and their domains are reported on the bottom and on the left of each matrix. Prp8 and Hsh155, divided in subdomains and motifs, respectively, are highlighted with boxes of different colors.

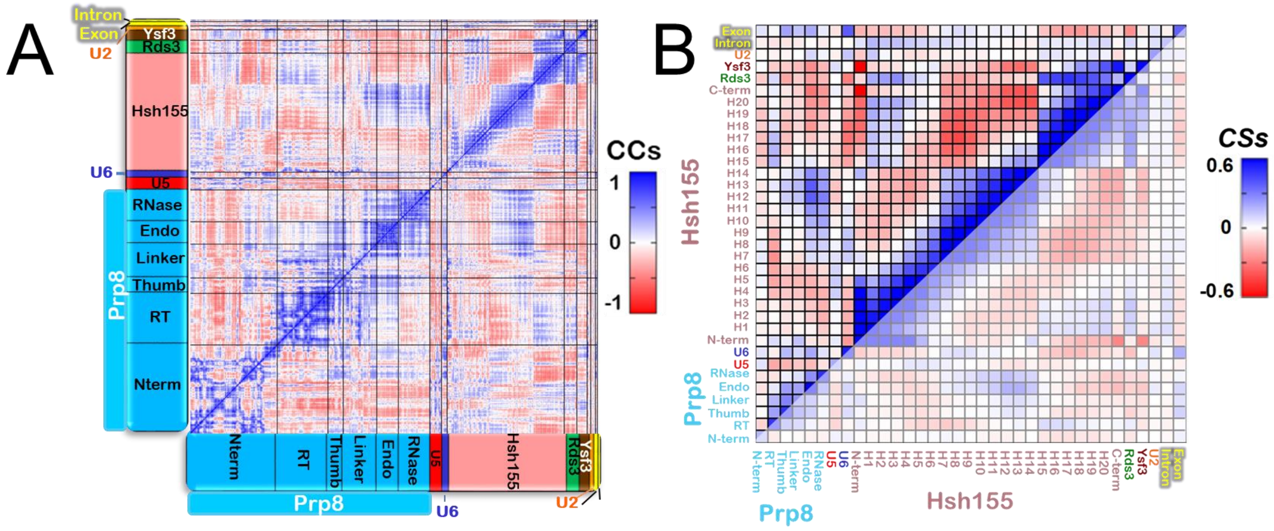

Figure S12. Monitoring convergence of cross correlation matrices. Cooperative motion leading the functional dynamics of the **Bact**. Per-residue Pearsons' cross coefficients (CCs) cross-correlation matrix derived from the mass-weighted covariance matrix. To evaluate convergence of the results this has been calculated over the last 380 ns of MD trajectories. (A) CCs values range from -1 (red, anti-correlated motions) to +1 (blue, correlated motions). (B) CCs values are summed for each pair of SPL proteins/domains, and normalized, as detailed in the Methods section of the main text, to provide a density correlation scores (CSs). CSs are here reported in the range from -0.6 to 0.6 for clarity reasons. The Hsh155 protein is here split by HEAT-repeat domains. The protein name and their domains are reported on the bottom and on the left of each matrix. Prp8 and Hsh155, divided in subdomains and motifs, respectively, are highlighted with boxes of different colors.

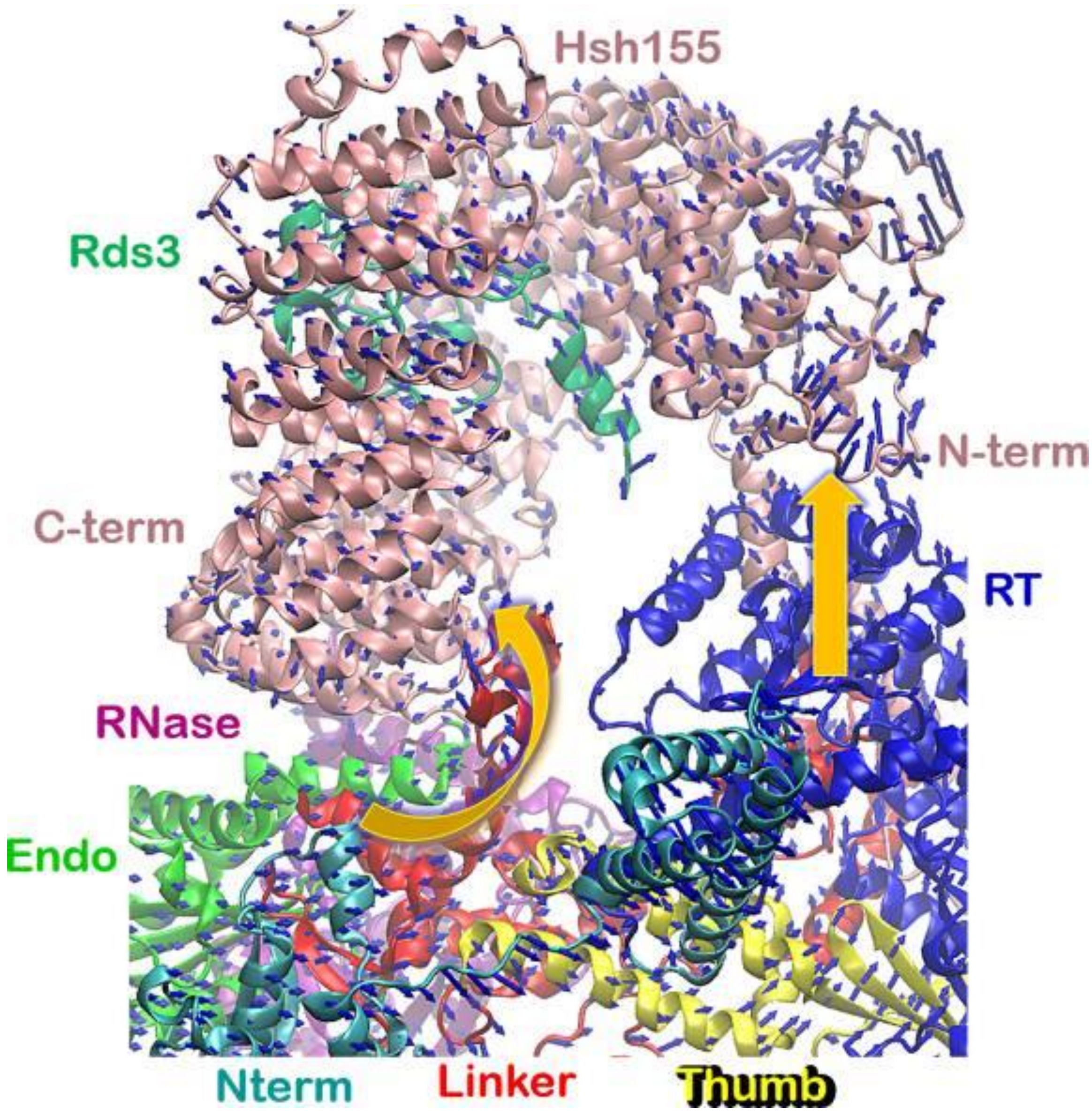

Figure S13. Essential dynamics of Bact. Essential dynamics of Hsh155, Rds3 and different domains of Prp8 as revealed by principal component analysis (PCA) of the **Bact** model. Blue arrows show the motion of Cα atoms, while the large orange arrows highlight the influence of distinct Prp8 domains on the global motion of Hsh155 and Rds3. All proteins are shown in new cartoon, with Hsh155 depicted in pink, and Rds3 in dark green. Prp8 domains Nterm, RT, Thumb, Linker, Endo and RNase are depicted in cyan, blue, yellow, red, light green, and purple, respectively.

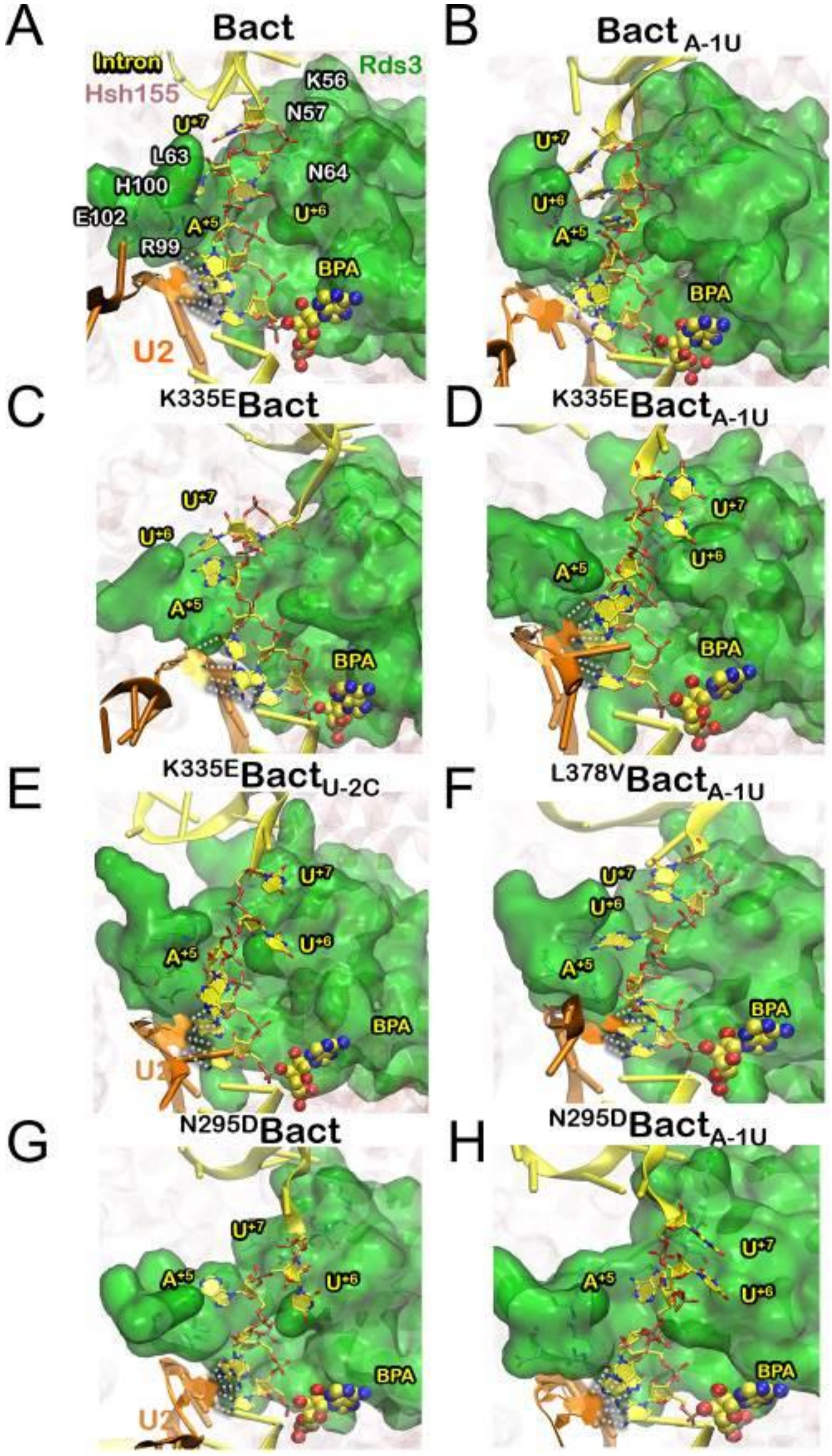

Figure S14. Close-up view of representative frames as extracted from the MD trajectories, depicting the intron (yellow) binding to Rds3 (green) for (A) **Bact**, (B) **Bact$_{\mathbf{A\text{-}1U}}$**, (C)

^{K335E}**Bact**, (D) ^{K335E}**Bact$_{A\text{-}1U}$**, (E) ^{K335E}**Bact$_{U\text{-}2C}$**, (F) ^{L378V}**Bact$_{A\text{-}1U}$,** (G) ^{N295D}**Bact,** and (H) ^{N295D}**Bact$_{A\text{-}1U}$** models. In the background Hsh155 is depicted in pale pink, BPA is represented in van der Waals spheres, intron is shown in licorice with C atoms colored in yellow, and U2 is represented in orange new cartoon. Hydrogen-bonds of base-pairs between intron and U2 are highlighted as white dashed lines. The Rds3 residues stabilizing the intron are labeled in white (A) and are omitted for clarity in (B) - (H).

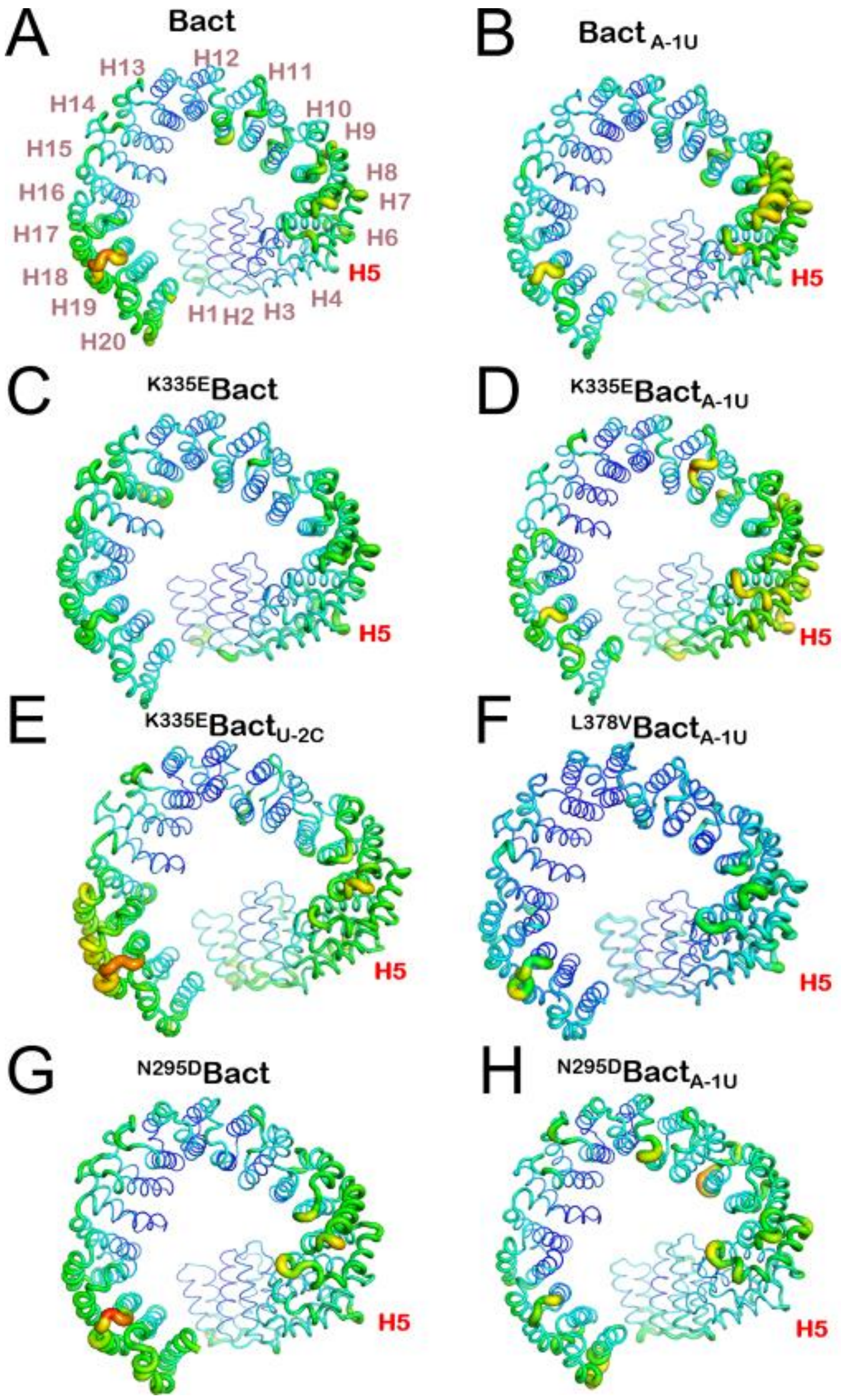

Figure S15. Root mean square fluctuations (RMSF) of Hsh155 protein depicted as B-factor representation (A) **Bact**, (B) **Bact$_{\mathbf{A\text{-}1U}}$**, (C) **$^{\mathbf{K335E}}$Bact**, (D) **$^{\mathbf{K335E}}$Bact$_{\mathbf{A\text{-}1U}}$**, (E) **$^{\mathbf{K335E}}$Bact$_{\mathbf{U\text{-}2C}}$**, (F) **$^{\mathbf{L378V}}$Bact$_{\mathbf{A\text{-}1U}}$,** (G) **$^{\mathbf{N295D}}$Bact,** and (H) **$^{\mathbf{N295D}}$Bact$_{\mathbf{A\text{-}1U}}$** models. N- and C-terminus of Hash155 are not shown for clarity.

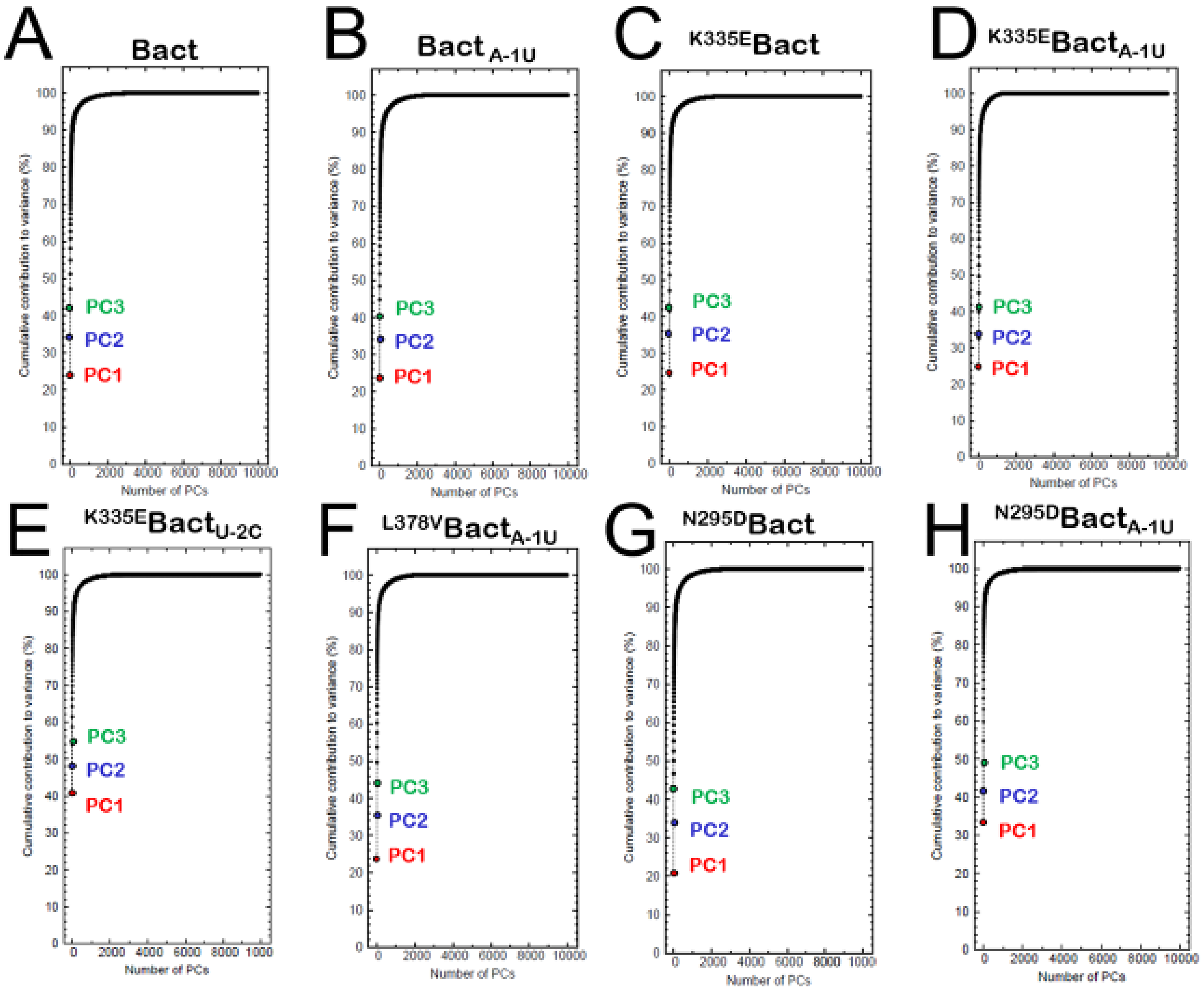

Figure S16. Principal components (PCs) cumulative contribution to variance for (A) **Bact**, (B) **Bact$_{A\text{-}1U}$**, (C) ^{K335E}**Bact**, (D) ^{K335E}**Bact$_{A\text{-}1U}$**, (E) ^{K335E}**Bact$_{U\text{-}2C}$**, (F) ^{L378V}**Bact$_{A\text{-}1U}$,** (G) ^{N295D}**Bact,** and (H) ^{N295D}**Bact$_{A\text{-}1U}$** models. On y-axis is depicted cumulative contribution of PCs (x-axis) to the variance of the overall motion calculated upon Principal Component Analysis. The contributions from the first three PCs are highlighted in red, blue and green, respectively.

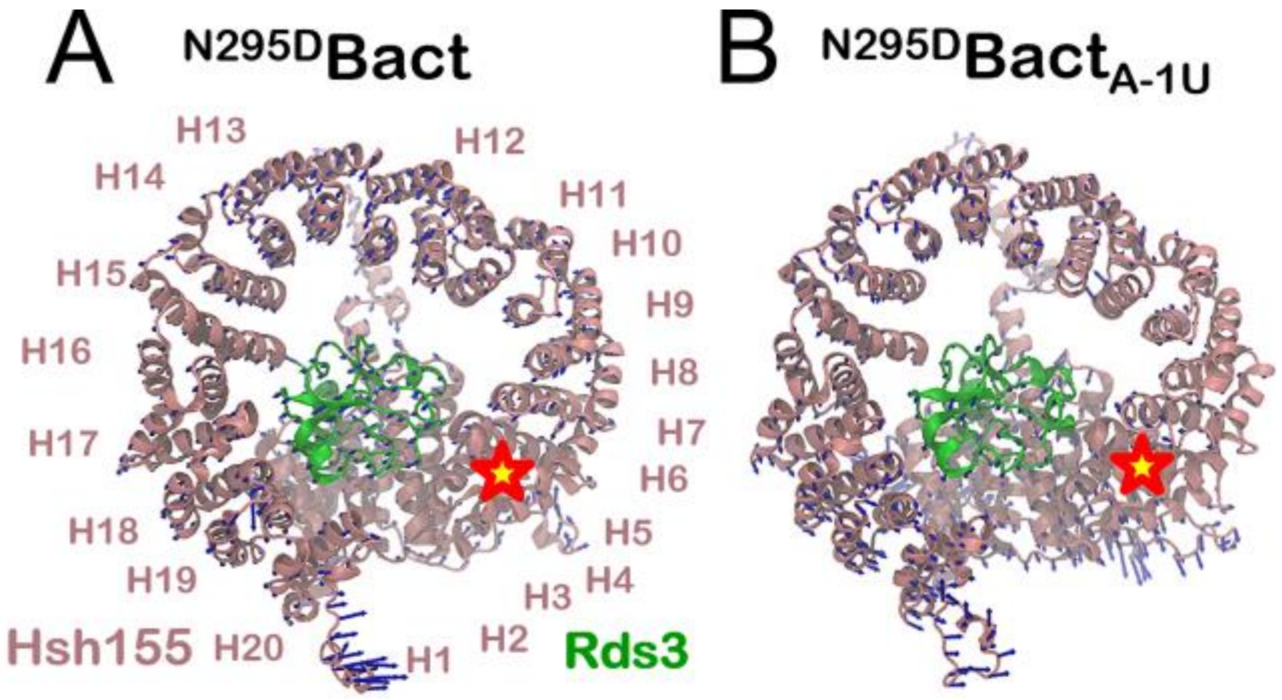

Figure S17. Essential dynamics as revealed by principal component analysis (PCA) for the Hsh155 protein in the (A) ^{N295D}**Bact,** and (B) ^{N295D}**Bact$_{A\text{-}1U}$** models. The (A) image shows the SF3b complex with Hsh155 protein labelled HEAT-repeats (pink), and Rds3 protein (green). The corresponding site of mutation is labelled as red star. Blue arrows are used to show the motion of Cα atoms along the first eigenvector.

Supplementary Tables

Table S1. Details of the proteins included in the spliceosome B^{act} model. Columns CHAIN, MOLECULE and RESOLUTION refer to original names and reported resolution from the original PDB structure (PDB ID 5GM6). Column MODELLED refers to de-novo modelled missing residues. Each considered molecule is highlighted with the same color as presented throughout the manuscript.

Spliceosome B^{act} model				
Cryo-EM 3.5 Å (5gm6)				
Total number of atoms (water included) = 666 641				
CHAIN	MOLECULE	CONSIDERED	MODELLED	RESOLUTION (Å)
A	Prp8	127-2085	432-450	2.8~4.0
D	U5 snRNA	[28-55] + [60-127]		2.8~4.0
E	U6 snRNA	41-85		2.8~4.0
G	Hsh155	16-971	75-155	3.0~3.5
J	Rds3	2-104		3.0~3.5
K	Ysf3	1-84		3.0~3.5
L	U2 snRNA	17-47		2.8~4.0
M	Intron	487-522		2.8~4.2
N	Exon	90-108		2.8~4.2
-	Mg+	#4		
-	ZNB	#3		
-	Na+	#167		
-	Wat	#603822		

Table S2. Occurrence of selected hydrogen (H)-bonds (reported as %) and average bond lengths (reported in Å) between the BPA and Hsh155 residues (A501, and K818, R775, Y826 and Q747), and residues in the vicinity of the mutation site (intron U^{+14} and Hsh155 residues X295, R299 and X335, where 'X' stands for the corresponding mutation) for the **Bact**, **Bact$_{A-1U}$**, **^{K335E}Bact**, **^{K335E}Bact$_{A-1U}$**, **^{K335E}Bact$_{U-2C}$**, **^{L378V}Bact$_{A-1U}$**, **^{N295D}Bact,** and **^{N295D}Bact$_{A-1U}$** models.

	BPA				mutation site			
**Model**	A501-K818 (% (Å))	A501-R775 (% (Å))	A501-Y826 (% (Å))	A501-Q747 (% (Å))	U^{+14}-R299 (% (Å))	X295-R299 (% (Å))	X335-R299 (% (Å))	X335-X295 (% (Å))
**Bact**	76 (2.83)	2 (3.01)	52 (2.65)	84 (2.99)	94 (2.86)	91 (2.86)	4 (2.93)	0
**Bact$_{A-1U}$**	83 (2.88)	1 (3.03)	68 (2.64)	73 (3.01)	92 (2.87)	89 (2.88)	0	0
**^{K335E}Bact**	79 (2.84)	9 (3.00)	72 (2.67)	65 (2.98)	97 (2.85)	90 (2.87)	0	7 (2.95)
**^{K335E}Bact$_{A-1U}$**	68 (2.86)	20 (2.97)	71 (2.68)	50 (3.02)	96 (2.86)	43 (2.90)	69 (2.88)	0
**^{K335E}Bact$_{U-2C}$**	73 (2.83)	1 (2.99)	53 (2.70)	47 (3.00)	95 (2.84)	47 (2.92)	64 (2.87)	0
**^{L378V}Bact$_{A-1U}$**	74 (2.85)	2 (3.01)	41 (2.71)	77 (2.97)	94 (2.85)	96 (2.84)	0	3 (2.98)
**^{N295D}Bact**	86 (2.84)	17 (2.98)	70 (2.64)	64 (3.04)	95 (2.89)	94 (2.86)	0	5 (3.01)
**^{N295D}Bact$_{A-1U}$**	86 (2.85)	1 (2.96)	26 (2.66)	71 (3.05)	96 (2.87)	95 (2.85)	0	79 (2.91)

Supplementary Movies

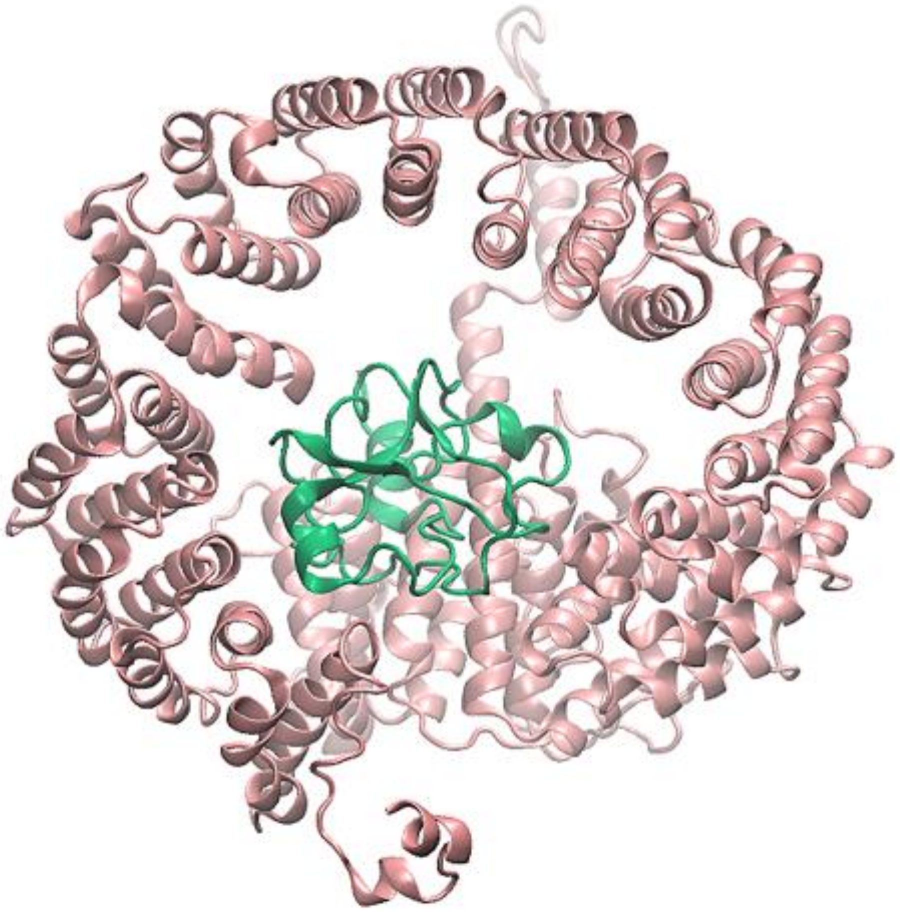

Movie S1. Principal Component Analysis (PCA) applied to Molecular Dynamics (MD) trajectories reveals the functional dynamics of Hsh155 (pink, new cartoon representation) and Rds3 (green, new cartoon representation) along the first eigenvector for the **Bact** model.

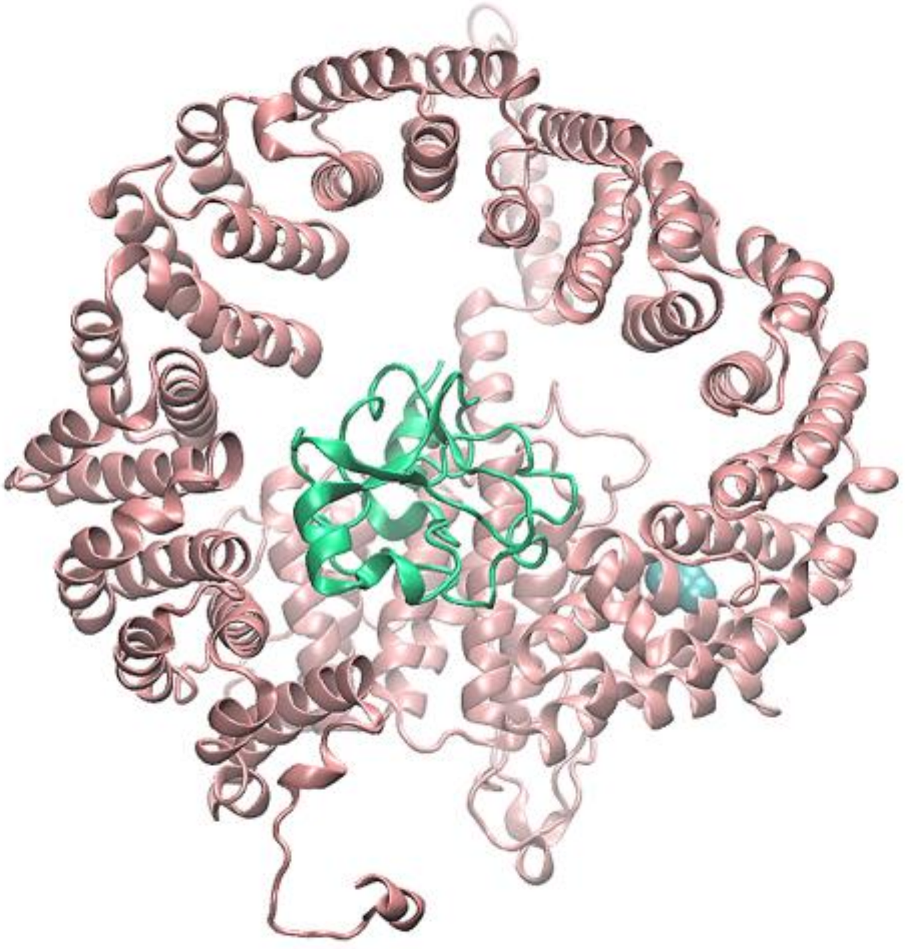

Movie S2. Principal Component Analysis applied to MD trajectories reveals the opening/closing motion of the Hsh155 (pink, new cartoon representation) and Rds3 (green, new cartoon representation) along the first eigenvector for the ^{K335E}**Bact**$_{A\text{-}1U}$ model. K335E mutation is shown in cyan van der Waals spheres.
